# Spiking neuron network Helmholtz machine

**DOI:** 10.3389/fncom.2015.00046

**Published:** 2015-04-21

**Authors:** Pavel Sountsov, Paul Miller

**Affiliations:** ^1^Neuroscience Graduate Program, Brandeis UniversityWaltham, MA, USA; ^2^Volen National Center for Complex Systems, Brandeis UniversityWaltham, MA, USA; ^3^Department of Biology, Brandeis UniversityWaltham, MA, USA

**Keywords:** spiking neural network, Bayesian inference, synaptic plasticity, unsupervised learning, sleep

## Abstract

An increasing amount of behavioral and neurophysiological data suggests that the brain performs optimal (or near-optimal) probabilistic inference and learning during perception and other tasks. Although many machine learning algorithms exist that perform inference and learning in an optimal way, the complete description of how one of those algorithms (or a novel algorithm) can be implemented in the brain is currently incomplete. There have been many proposed solutions that address how neurons can perform optimal inference but the question of how synaptic plasticity can implement optimal learning is rarely addressed. This paper aims to unify the two fields of probabilistic inference and synaptic plasticity by using a neuronal network of realistic model spiking neurons to implement a well-studied computational model called the Helmholtz Machine. The Helmholtz Machine is amenable to neural implementation as the algorithm it uses to learn its parameters, called the wake-sleep algorithm, uses a local delta learning rule. Our spiking-neuron network implements both the delta rule and a small example of a Helmholtz machine. This neuronal network can learn an internal model of continuous-valued training data sets without supervision. The network can also perform inference on the learned internal models. We show how various biophysical features of the neural implementation constrain the parameters of the wake-sleep algorithm, such as the duration of the wake and sleep phases of learning and the minimal sample duration. We examine the deviations from optimal performance and tie them to the properties of the synaptic plasticity rule.

## 1. Introduction

Humans and other animals live in a predictable and structured environment where they are required to make rapid and effective decisions in order to procure food, escape predators, and find mates. These sensory inputs, however, provide only a limited and often corrupted snapshot of the environment around the animal. Although decisions are made using this imperfect information, they must reflect the actual nature of the environment, as it is that which determines the effect of an animal's action.

Bayesian inference provides the mathematical description of how to make optimal decisions given this limited and corrupted information about the environment (Bishop, 2006; Griffiths et al., 2010). There is ample experimental data showing that humans and other animals behave in a way consistent with Bayesian inference in probabilistic tasks such as cue combination (van Beers et al., 1999; Atkins et al., 2001; Ernst and Banks, 2002; Alais and Burr, 2004; Burge et al., 2010), combination of uncertain evidence with prior knowledge (Tassinari et al., 2006), sensory-motor learning (Körding and Wolpert, 2004), motion illusions (Weiss et al., 2002), and causal reasoning (Blaisdell et al., 2006). Optimal learning on lifetime (Griffiths and Tenenbaum, 2006) and experimental (Orbán et al., 2008; Chalk et al., 2010) timescales has also been observed. Analysis of neural recordings during perception of natural scenes throughout development is also broadly consistent with optimal inference (Berkes et al., 2011). This evidence motivates the search for an implementation of Bayesian inference in the brain.

**Figure 1:**
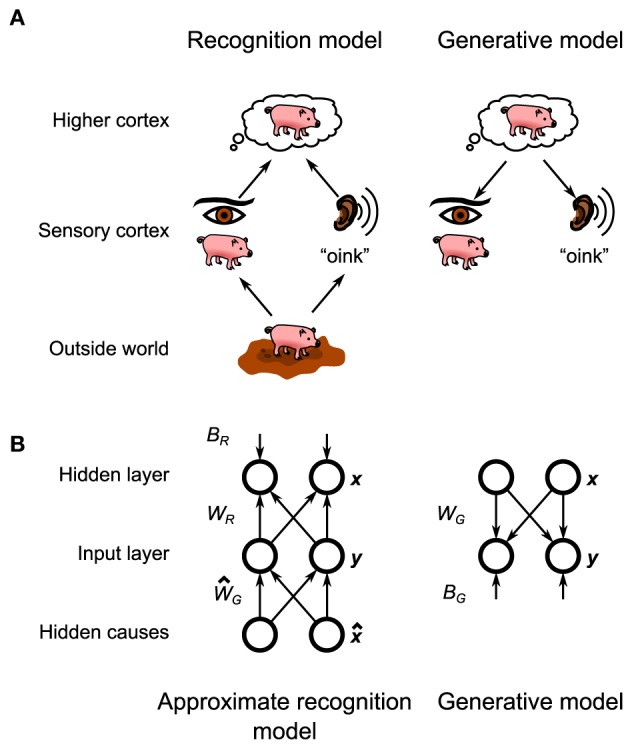
**General layout of the Helmholtz Machine. (A)** The putative Helmholtz Machine in the brain consists of two separate models, the recognition model and the generative model. The recognition model transforms the neural activity in the early sensory cortices (set up by the objects in the outside world) to set up neural activity in the higher cortices, which represents the inferred structure of the outside world. The generative model goes in reverse, transforming the neural activity in the higher cortices (set up by top-down connections from even higher cortices) into neural activity in the sensory cortices, which represents the reconstructed sensory stimulus that corresponds to the world fantasized by the higher cortex. **(B)** The Helmholtz Machine in this paper consists of two input nodes and two hidden nodes. The model learns the recognition weights, *W*_*R*_, and biases, *B*_*R*_, as well as the generative weights, *W*_*G*_, and biases, *B*_*G*_.

In this framework the brain holds a probabilistic model of the physical laws which translate the makeup of the environment (e.g., the objects that are in front of the animal) into the (corrupted) sensory information that enters the brain (Figure 1A, bottom). As originally posed by Helmholtz (1925) the brain then inverts this probabilistic model, also called the generative model, to create a recognition model, that converts that corrupted information into an optimal estimate of the makeup of the environment that the brain can then make decisions about (Figure 1A, top). Learning in this framework involves adjusting the parameters of the generative model (and thus its inverse, the recognition model) to match the statistics of the environment. As exact Bayesian inference is typically intractable (Bishop, 2006), an approximate recognition model is often required. This approximation would manifest itself in the animal's behavior in the form of specific behavioral biases (Sanborn et al., 2010).

We are interested in examining Bayesian inference at the level of neural implementation. This has two benefits. First, the neural substrate adds an additional level of approximation and bias which may aid the interpretation of behavioral data, as above. Secondly, a neural specification of an algorithm will specify the type of data that should be looked for in neural recordings. There have been multiple proposals of how to implement Bayesian inference in neuronal networks (Lee, 2002; Friston and Kiebel, 2009; Moran et al., 2013) and some have advanced to using spiking neurons (Rao, 2005; Ma et al., 2006, 2008; Shi and Griffiths, 2009; Buesing et al., 2011; Pecevski et al., 2011). Most of these past attempts did not address the question of learning. More recently, proposals to implement both inference and learning in model neurons with both a stochastic (Brea et al., 2011; Rezende et al., 2011; Nessler et al., 2013) and deterministic (Deneve, 2008a,b) spiking mechanism have been developed. We propose an alternative formulation (detailed below) based on neurons with deterministic dynamics with the required stochasticity originating from stochastic release of in synaptic vesicles.

In this paper we will explore the questions of both inference and learning by providing a spiking neuron model implementation of a particular algorithmic model of Bayesian inference called the Helmholtz machine. The Helmholtz machine (Dayan et al., 1995; Hinton et al., 1995; Hinton and Dayan, 1996; Neal and Dayan, 1997; Dayan, 1999, 2000) provides a method of performing both approximate inference and learning in a way that is amenable to biological implementation, because unlike similarly powerful models, connection-strength changes depend only upon local correlations. In addition to the recognition model common to all implementations of Bayesian inference, the Helmholtz machine posits the existence of an explicit generative model in the brain (Figure 1A). This generative model is not used during inference, but is critical for learning the parameters of the recognition model (the recognition model, in turn, is used to train the parameters of the internal generative model). The original Helmholtz machine was successfully tested as a model of handwritten digit recognition (Hinton et al., 1995) and factor analysis (Neal and Dayan, 1997). The details of biological implementation of these ideas have hitherto been incomplete, with the issue of how to implement its learning rule, the delta rule, being particularly vexing. The proposed model will show how a microcircuit combined with a experimentally observed synaptic plasticity rule can implement the required computations to bridge the gap between the algorithm and the neural substrate.

## 2. Materials and methods

### 2.1. Computational Helmholtz machine model

#### 2.1.1. Model description

The generative model that we will be implementing and using to model (simulated) observed data is a mixture model of truncated gaussians:

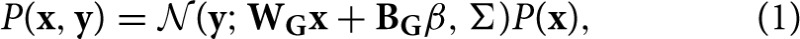



where **x** is the activity of the units in the hidden layer and **y** is the activity of the units in the observed layer. A truncated gaussian is given by the following probability distribution function:

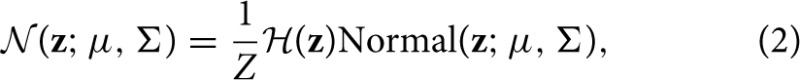



for some mean μ, covariance matrix Σ and normalizing coefficient *Z*. 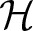
(**z**) is a multivariate Heaviside function:

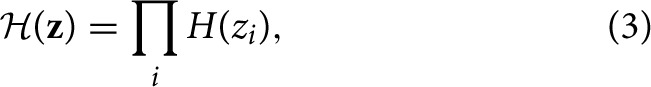



where *H*(·) is the usual Heaviside function (with *H*(0) = 1) and the product is taken over all components of **z**.

The goal of perception as formulated in the Bayesian framework is to invert this generative model, i.e., to find *P*(**x**|**y**). This is intractable in the general case, so an approximate recognition model is used. In this case the approximate recognition model is also a mixture of truncated gaussians:

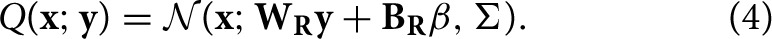



Both model share a fixed bias activity β and the variance matrix **Σ**. The remaining parameters, namely the generative weights **W**_**G**_, biases **B**_**G**_, recognition weights **W**_**R**_, and biases **B**_**R**_ are learned by the model using the wake-sleep algorithm. The values of the fixed parameters as well as the initial values of the learned parameters are detailed in Table S1. Since the variance parameters are not learned, the exact functional form of the mixture components does not significantly matter (e.g., a Poisson distribution would result in an identical neural implementation) as long as its mean depends linearly on the weights and biases. We choose a truncated gaussian for mathematical convenience.

#### 2.1.2. Learning rules

It is possible to derive the exact wake-sleep learning rules for truncated gaussian units by following a standard procedure (see Supplementary Information). During the wake phase of the algorithm the generative model is adapted to the environment by first estimating the hidden layer activities **x**^(*n*)^ given an observation **y**^(*n*)^ from the environment using the approximate recognition model and then adjusting the generative weights and biases as follows:

(5) $$\Delta W_{Gij}=\eta x_{j}^{\left(n\right)}\left(y_{i}^{\left(n\right)}-\left(W_{G}x^{\left(n\right)}+B_{G}\beta \right)\right)$$

(6) $$\;\Delta B_{Gi}=\eta b\left(y_{i}^{\left(n\right)}-\left(W_{G}x^{\left(n\right)}+B_{G}\beta \right)\right),$$

where η is the learning rate. During the sleep phase the approximate recognition model is adapted to better invert the generative model by first generating a sample {**x**^(*n*)^, **y**^(*n*)^} from the generative model and then adjusting the recognition weights and biases:

(7) $$\Delta W_{Rij}=\eta y_{j}^{\left(n\right)}\left(x_{i}^{\left(n\right)}-\left(W_{R}y^{\left(n\right)}+B_{R}\beta \right)\right)$$

(8) $$\;\Delta B_{Ri}=\eta b\left(x_{i}^{\left(n\right)}-\left(W_{R}y^{\left(n\right)}+B_{R}\beta \right)\right).$$

During learning we constrain **B**_**R**_ and **B**_**G**_ to be positive while **W**_**G**_ and **W**_**R**_ are not constrained.

**Figure 2:**
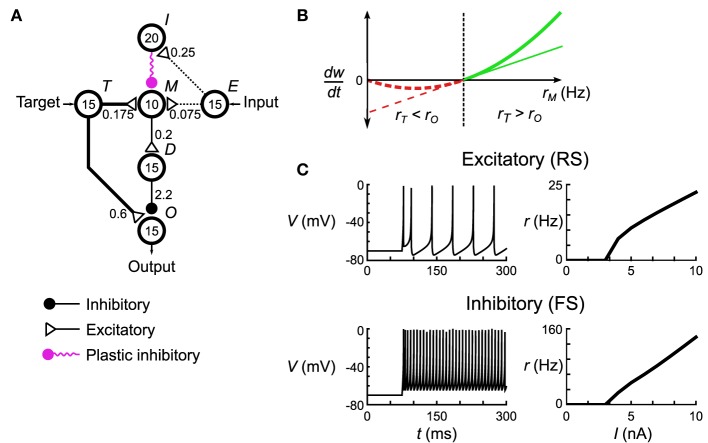
**Spiking neuronal network implementation of the delta rule. (A)** The microcircuit implementing the delta rule consists of interconnected pools of neurons (circles). Numbers in the circles signify the number of neurons in each pool. Numbers next to the connections indicate maximum total conductance through that pathway in nS. The connections have a sparsity of *s* = 0.3. Not shown are the non-specific external connections made to each neuron in pools *M* and *O*. Each individual post-synaptic neuron in those pools gets its own Poisson spike train with rate *r*_ϵ_ = 100 Hz that excites it through an excitatory synapse with conductances of 0.42 nS and 0.6 nS, respectively. **(B)** The spiking plasticity rule at the plastic inhibitory synapses implements a form of the BCM rule. Synapses depress (dashed red lines) or potentiate (solid green lines) depending on whether the post-synaptic firing rate, *r*_*M*_, is respectively less than (or greater than) some threshold rate *r*_θ_ (thick line). By controlling *r*_*M*_ to be below *r*_θ_ when the delta rule (thin line) predicts depression, and vice versa when the delta rule predicts potentiation, this microcircuit approximates the delta rule. **(C)** Responses of the model neurons to DC current injection. Once the neurons exceed a certain threshold current, their firing rate is approximately linear, which leads to an overall linearity of this microcircuit.

Our neuronal network model will implement the above learning rules by approximating them using biologically plausible synaptic plasticity rules (Figure 2). To focus our analysis on the difference between the computational implementation and the implementation using the neural substrate we also use an approximate learning rule in the computational model (i.e., we do not use Equations 5–8). Given a target activity *T*, an input activity *I* and an output activity *O*, let the error signal be *M* = max(*T* − *O*, −θ). The weight is then adjusted as follows:

(9) Δw=ηIM(M+θ),

where θ is some threshold activity. Equations (5, 6), for example, are approximated by

$$\begin{array}{l} \Delta W_{Gij}=\eta x_{j}^{\left(n\right)}M(M+\theta )\\ \;\Delta B_{Gi}=\eta bM(M+\theta )\\ \;M=\text{max}\left(y_{i}^{\left(n\right)}-\left(W_{G}x^{\left(n\right)}+B_{G}\beta \right),-\theta \right). \end{array}$$

This approximation can be derived by assuming that the unit activity is encoded using Poisson spike trains and that the connection weights are adjusted using realistic synaptic plasticity rules (see Supplementary Information). The quality of this approximation is verified empirically.

### 2.2. Neuronal network delta rule model

#### 2.2.1. Neural model and synaptic currents

To simulate spiking neurons we use a two variable model spiking neuron introduced by Izhikevich (2003). The model is described by two coupled ordinary differential equations and a single threshold condition:

(10) $$\frac{dV}{dt}=0.04V^{2}+5V+140-u+I_{e}+I_{i}+I_{r}$$

(11) $$\frac{du}{dt}=a(bV-u)$$

(12) $$\text{if }V>0\text{ mV}\to \{\begin{array}{l} V\mapsto c\\ u\mapsto u+d \end{array}\;,$$

where *V* is the membrane potential (measured in mV), *u* is an adaptation current, *a*, *b*, *c*, *d* are the parameters that determine the dynamics of the neural firing. We use two sets of parameters to model excitatory (regular spiking—RS) neurons and inhibitory (fast spiking—FS) neurons (Table S2).

*I*_*e*_ models the excitatory current flowing through AMPA and NMDA receptors:

$$I_{e}=(g_{e1}+g_{e2}\text{NMDA}(V))(E_{e}-V),$$

where *g*_*e*1_ and *g*_*e*2_ are the summed dynamic conductances (measured in nS) of all excitatory synapses onto a neuron. NMDA(*V*) describes the membrane potential dependence of the NMDA current (Jahr and Stevens, 1990):

$$\text{NMDA}(V)=\frac{1}{1+\frac{{\left[{\text{Mg}}^{++}\right]}_{\text{ext}}}{3.57}\exp(-0.062V)}.$$

*I*_*i*_ models the inhibitory current flowing through GABA_A_ channels:

$$I_{i}=g_{i}(E_{i}-V),$$

where *g*_*i*_ is the summed dynamic conductance (measured in nS) of all inhibitory synapses onto a neuron.

*I*_*r*_ is a current coming from excitatory synapses (modeled as AMPA currents) that are external to the network:

$$I_{r}=g_{r}(E_{e}-V),$$

where *g*_*r*_ is the dynamic conductance (measured in nS) of of these external synapses.

Whenever a non-external pre-synaptic neuron fires, after a certain delay Δ, the conductance of the corresponding synapse type gets adjusted by a random amount:

$$g_{syn}\mapsto g_{syn}+w\text{ max}(f,0),$$

where *w* is the synaptic weight (measured in nS) and *f* is the number of vesicles released during the event. The distribution of *f* is modeled by a binomial distribution (Castillo and Katz, 1954) with a fixed number of vesicles, *N*_*v*_, and the probability of release, *P*_*v*_ (Table S2). For computational convenience, we approximate this binomial distribution by a normal distribution with mean *N*_*v*_*P*_*v*_ and variance *N*_*v*_*P*_*v*_(1 − *P*_*v*_) truncated at 0.

The external synapses are modeled as a Poisson process with rate *r*_*r*_ Hz. Whenever an external “neuron” fires, the conductance *g*_*r*_ gets adjusted by a fixed amount *w*_*r*_.

Between spiking events all of the aforementioned conductances evolve as ordinary first order differential equations:

$$\begin{array}{l} \tau_{e1}\frac{dg_{e1}}{dt}=-g_{e1}\\ \tau_{e2}\frac{dg_{e2}}{dt}=-g_{e2}\\ \;\tau_{i}\frac{dg_{i}}{dt}=-g_{i}\\ \;\tau_{e1}\frac{dg_{r}}{dt}=-g_{r}. \end{array}$$

#### 2.2.2. Network connectivity

The delta rule networks are subdivided into homogeneous pools of neurons, identified by labels (Table S3, Figure 2). Each network has an input pool *E*, an output pool *O* and a target pool *T* which are used to provide inputs to and read outputs from the network. Additionally, there are intermediate pools *M* and *D* used by the network to perform computation and learning (see Results). Pairs of neuronal pools are connected with directed, sparse connections (Table S4). Each neuron in the source pool is connected with the same fraction of neurons in the destination pool. The total conductance of synapses made by a pre-synaptic neuron is fixed, with each synapse getting an equal portion of that total. The connection sparsity, *s*, is the same across all inter-pool connections.

#### 2.2.3. Synaptic plasticity

The Spiking BCM synaptic plasticity rule estimates the post-synaptic spike rate $\hat{r}$_*post*_ (measured in Hz) by filtering the post-synaptic spike train using an exponential kernel:

$$\begin{array}{l} \;\kappa (t)=\{\begin{array}{ll} 0 & \text{if }t<0\\ \frac{1}{\tau }\exp\left(-\frac{t}{\tau }\right) & \text{if }t\geq 0 \end{array}\\ {\hat{r}}_{post}(t)=\sum_{j}\kappa (t-t_{j}), \end{array}$$

where *t*_*j*_ is the time of *j*'th post-synaptic spike. During every pre-synaptic event (that happens at time *t*_*i*_ for *i*'th pre-synaptic spike) the synaptic weight gets adjusted as follows:

$$w\mapsto w+A{\hat{r}}_{post}(t_{i})({\hat{r}}_{post}(t_{i})-r_{\theta }).$$

The STDPi synaptic plasticity rule computes an estimate of both the pre-synaptic spike rate $\hat{r}$_*pre*_ and the post-synaptic spike rate $\hat{r}$_*post*_ (both measured in Hz) by filtering the pre- and post-synaptic spike trains respectively using a difference-of-exponentials kernel:

$$\begin{array}{l} \;\kappa (t)=\{\begin{array}{ll} 0 & \text{if }t<0\\ \frac{1}{\tau_{1}\;-\;\tau_{2}}\left(\exp\left(-\frac{t}{\tau_{1}}\right)-\exp\left(-\frac{t}{\tau_{2}}\right)\right) & \text{if }t\geq 0 \end{array}\\ {\hat{r}}_{pre}(t)=\sum_{i}\kappa (t-t_{i})\\ {\hat{r}}_{post}(t)=\sum_{j}\kappa (t-t_{j}), \end{array}$$

where *t*_*i*_ is the time of *i*'th pre-synaptic spike and *t*_*j*_ is the time of *j*'th post-synaptic spike. During each pre-synaptic event the synaptic weight gets adjusted as follows:

(13) $$w\mapsto w-A_{m}{\hat{r}}_{post}(t_{i})$$

During each post-synaptic event the synaptic weight gets adjusted as follows:

(14) $$w\mapsto w+A_{p}{\hat{r}}_{pre}(t_{j}){\hat{r}}_{post}(t_{j}).$$

The plastic weights are restricted to be non-negative.

To examine the properties of the two plasticity rules we construct two artificial spike trains. The pre-synaptic spike train is created using a homogeneous Poisson process with a constant rate *r*_*pre*_. The post-synaptic spike train is created using an inhomogeneous Poisson process with a post-synaptic spike rate *r*_*post*_(*t*):

$$\begin{array}{l} r_{post}(t)=\max\left(0,r_{base}+\Delta r\sum s(t-t_{i})\right)\\ \;s(t)=\{\begin{array}{ll} 0 & \text{if }t<0\\ \exp\left(-\frac{t}{\tau_{r}}\right) & \text{if }t\geq 0 \end{array}, \end{array}$$

where *t*_*i*_ is the time of the *i*'th pre-synaptic spike. When plotting, we use *r*_*post*_, which is the mean across time of *r*_*post*_(*t*). We vary *r*_*base*_ to produce the necessary *r*_*post*_. These parameters are listed in Table S1.

#### 2.2.4. Training and testing protocol

To set the target and input rates we vary *r*_*r*_ for neurons inside the pools *T* and *E*, respectively. We keep the rate constant throughout the training and testing periods. The output rate is computed by averaging the firing rate of all neurons inside output pool *O* during the testing duration. During the testing period the plasticity is turned off (e.g., by setting the appropriate plasticity change amplitudes to 0).

### 2.3. Neuronal network Helmholtz machine model

#### 2.3.1. Network connectivity

The neuronal network implementation of the Helmholtz Machine is constructed out of four units, arranged in two layers. These units correspond exactly to the computational model units **x** and **y**. Each unit has the same connectivity as the delta rule network except that pool *E* is removed and pool *R* and the variable labeled output pools *X*_1_, *X*_2_, *Y*_1_, *Y*_2_ are added (Table S3). Additionally, pool *I* is now associated with the source pool and not with the destination pool (although connectivity is the same). These subunits are interconnected by making excitatory projections from the output pool and inhibitory projections from pool *I* of a unit on one layer to pool *M* of a unit in a different layer (Table S4).

#### 2.3.2. Training and testing protocol

The inputs into the network come entirely through setting the *r*_*r*_ variable of pool *T* to *r*_*T*_. During the wake phase, *r*_*T*_ is set to *y*^(*n*)^_1_ and *y*^(*n*)^_2_ for units *y*_1_ and *y*_2_ and to *r*_*ns*_ for units *x*_1_ and *x*_2_. During sleep phase the inverse happens: *r*_*T*_ is set to *x*^(*n*)^_1_ and *x*^(*n*)^_2_ for units *x*_1_ and *x*_2_ and to *r*_*ns*_ for units *y*_1_ and *y*_2_. Additionally, the connectivity between units changes between phases, as detailed in Table S4.

The probability distributions over the output rates are computed by computing a histogram of samples collected from the network. A sample is computed by averaging the rate of all neurons in the relevant output pool for the duration of the sample, *T*_*sample*_.

### 2.4. Prior distributions

The generative tests and the reward test use priors coming from two families of unimodal and bimodal priors:

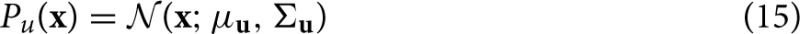

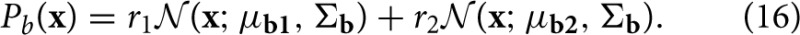





The parameters used for these prior distributions are listed in Table S5.

The decoding test uses a uniform line prior:

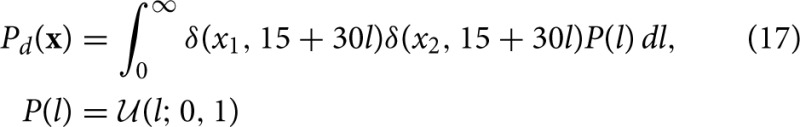



where 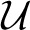
(*z*; *a*, *b*) is the uniform distribution sampling *z* from [*a*, *b*] and δ(·, ·) is the Kronecker delta. When plotting, we collect *N*_*test*_ samples from each distribution.

### 2.5. Training data sets

The computational model and the neuronal network are trained on a set of 10 data sets, with the first eight used for the generative model tests and the last two for the decoding test and the reward test respectively. The data sets are generated prior to training (and are reused for all models) by drawing *N*_*test*_ samples from the probability distributions. We chose the training distributions such that they avoided low firing rates where our learning rules have the most inaccuracy.

During training the data points are taken successively and in the same order for all trials (restarting from the beginning when the data set is exhausted). The unimodal data sets *a* … *e* are drawn from a truncated gaussian distribution: 
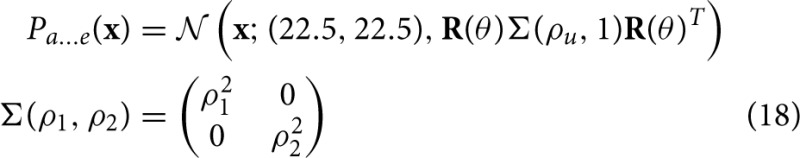

(19) $$R(\theta )=\left(\begin{array}{cc} \cos(\theta ) & -\sin(\theta )\\ \sin(\theta ) & \cos(\theta ) \end{array}\right).$$

The bimodal data sets *f* … *i* are drawn from a mixture of two truncated gaussians:

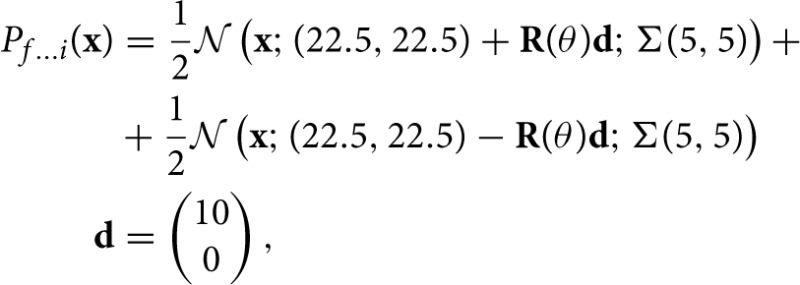



where **Σ**(ρ_1_, ρ_2_) and **R**(θ) are defined in Equations (18, 19). The parameters for these distributions are detailed in Table S6.

The data set, *o*, for the decoding task is drawn from a uniform line distribution with added truncated gaussian noise:

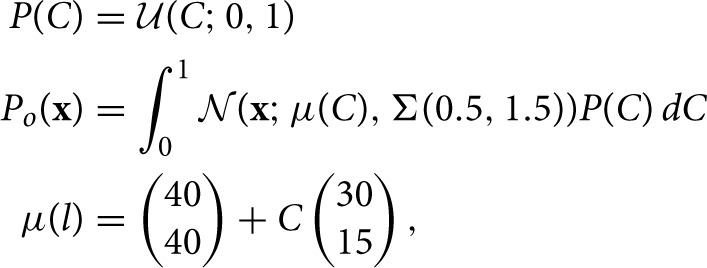



where 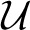
(*z*; *a*, *b*) is the uniform distribution sampling *z* from [*a*, *b*] and **Σ**(ρ_1_, ρ_2_) is defined in Equation (18). The data points are labeled by the value of the variable *C* used to generate them.

The data set, *r*, for the reward task is drawn from a uniform line distribution:

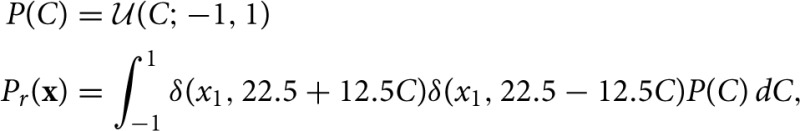



where δ(·, ·) is the Kronecker delta. The data points are labeled by the value of the variable *C* that was used to generate them.

### 2.6. Generative model test

We compute the similarity between two probability distributions *P*(*x*) and *Q*(*x*) using the Jensen-Shannon divergence (Lin, 1991):

$$\begin{array}{l} D(P∥Q)=\frac{1}{2}D_{KL}(P∥M)+\frac{1}{2}D_{KL}(Q∥M)\\ \;M(x)=\frac{P(x)+Q(x)}{2}, \end{array}$$

where *D*_*KL*_ is the Kullback–Leibler divergence (Kullback and Leibler, 1951):

$$D_{KL}(P∥Q)=\int_{-\infty }^{\infty }P(x)\log_{2}\left(\frac{P(x)}{Q(x)}\right)\;dx.$$

We use base 2 for the logarithm so that *D* ranges from 0 to 1. Since we, in most cases, do not have access to the complete probability distributions, we estimate *D* by computing it between two 2D histograms. The histograms are 40 bins on each side (total of 1600 bins) and range from 0 Hz to 60 Hz. This method of computing *D* is biased (Treves and Panzeri, 1995), but it is sufficiently accurate for the purposes of this paper.

### 2.7. Decoding test

Given a sample **x**^(*n*)^ from the computational model or from a neuronal network we decode the represented value ${\hat{C}}^{\left(n\right)}$ by projecting that sample onto the line formed by the prior:

$${\hat{C}}^{\left(n\right)}=\frac{\left(x_{1}^{\left(n\right)}-15)+(x_{2}^{\left(n\right)}-15\right)}{30}.$$

We can then compute the mean deviation like so:

$$\text{MeanDev}=\sqrt{\frac{\sum {\left({\hat{C}}^{\left(n\right)}-C^{\left(n\right)}\right)}^{2}}{N_{test}}},$$

where *C*^(*n*)^ is the label associated with **y**^(*n*)^ which was drawn from the data set and that generated **x**^(*n*)^.

### 2.8. Reward test

We decode the represented value ${\hat{C}}^{\left(n\right)}$ using a method similar to that for the decoding test (except with a different prior):

$${\hat{C}}^{\left(n\right)}=\frac{-x_{1}^{\left(n\right)}+x_{2}^{\left(n\right)}}{25}.$$

We then compute the response probability, $P\left({\hat{C}}^{\left(n\right)}>0|C^{\left(n\right)}\right)$, of the model and fit it with a logistic function:

(20) $$P\left({\hat{C}}^{\left(n\right)}>0|C^{\left(n\right)}\right)=\frac{1}{1+\exp\left(-\frac{C^{\left(n\right)}-\mu }{s}\right)}.$$

We interpret μ as the decision threshold and *s* as a measure of the internal system noise.

We compute the reward attained by the model as follows:

$$\begin{array}{l} \;R=\frac{1}{N_{test}}\sum r_{1}S\left({\hat{C}}^{\left(n\right)}>0,C^{\left(n\right)}>0\right)\\ \;+r_{2}S\left({\hat{C}}^{\left(n\right)}<0,C^{\left(n\right)}<0\right)\\ S(a,b)=\{\begin{array}{ll} 1 & \text{if }a=\text{True},\;b=\text{True}\\ 0 & \text{otherwise} \end{array}\;, \end{array}$$

where *r*_1_ and *r*_2_ are the rewards associated with the two choices. Since there is a symmetry in the Helmholtz Machine, we flip *x*_1_ and *x*_2_ so as to switch the interpretation of all trials, if such a flip increases the computed reward.

If we assume that the system noise is distributed as a logistic distribution, then given the decision threshold μ and internal system noise scale *s* the reward attained by a noisy decoder is:

(21) $$\begin{array}{l} R_{logistic}(\mu ,s)=\;\frac{1}{2}r_{1}\int_{0}^{1}\int_{\mu }^{\infty }\text{Logistic}(m;C,s)\;dm\;dC\\ \;+\;\frac{1}{2}r_{2}\int_{-1}^{0}\int_{-\infty }^{\mu }\text{Logistic}(m;C,s)\;dm\;dC. \end{array}$$

The optimal threshold μ_*opt*_ is obtained by maximizing *R*_*logistic*_:

$$\begin{array}{l} \;\mu_{opt}=\underset{\mu }{\arg\max}R_{logistic}(\mu ,s)\\ R_{max}(s)=R_{logistic}(\mu_{opt},s), \end{array}$$

where the noise scale is estimated from the model for trials where *r*_1_ = *r*_2_. The minimal reward that can be obtained given our scoring methodology is:

$$R_{min}=\frac{\max(r_{1},r_{2})}{2},$$

which can also be obtained by computing the appropriate limit of Equation (21).

This task is particularly prone to convergence issues so we discarded models which had decision thresholds outside the range [*Q*1 − 1.5*IQR*, *Q*3 + 1.5*IQR*], where *Q*1 and *Q*3 are the first and third quartiles and *IQR* = *Q*3 − *Q*1.

### 2.9. Computer simulations

The computational model was simulated using custom code written in the D programming language (Alexandrescu, 2010) and run on the authors' personal computer. The neuronal networks were simulated using a neural simulator written in the D programming language by the authors. The simulator uses OpenCL (Stone et al., 2010) to run on both GPGPU resources (AMD Radeon HD 5830 on the authors' personal computer) and CPU resources (High Performance Computing Cluster at Brandeis University). Source code for all of the models is available on author's webpage.

Model fitting for the reward test was done using the SciPy package (Jones et al., 2001) and the Python programming language (van Rossum and Drake, 2001).

## 3. Results

### 3.1. Computational Helmholtz machine model

The specific Helmholtz Machine implemented in this paper consists of two observed units **y** and two hidden units **x** (Figure 1B). Here, “observed” means that this unit directly receives sensory data from the environment (thus observing the environment) while “hidden” means that it does so indirectly (i.e., the environment is hidden from it). In practical terms the observed units can model the early sensory areas of the brain, while hidden units can model later sensory areas. Each unit codes for a single continuous non-negative quantity. The generative model and the approximate recognition model are parameterized by generative weights, generative biases, recognition weights, and recognition biases.

Weights and biases are learned using an unsupervised learning algorithm called the wake-sleep algorithm (Hinton et al., 1995). It consists of two quasi-symmetrical phases: wake and sleep. During the wake phase, samples **y**^(*n*)^ are taken from the training data set and are used to generate samples **x**^(*n*)^ from the approximate recognition model. The generative weights and biases are then used to reconstruct the training data, with the error in reconstruction used to adjust the generative weights. In our model, the rule used for this purpose is the delta rule:

(22) $$\Delta w=\eta z_{I}(z_{T}-z_{O}),$$

where *z*_*I*_ is the value of the input unit (in this case one of the hidden units), *z*_*O*_ is the value of the output unit (in this case the reconstructed training data for one of the observed units) and *z*_*T*_ is the target value (in this case the true value of the training data). *w* can either be a weight between two units in the different layers (in this case the top-down generative weight) or a bias weight, in which case the value of the input unit is taken to be β. During the sleep phase samples **x**^(*n*)^ are taken from the prior distribution (equivalently they are taken from the distribution on the hidden units conditioned on the activity of higher brain areas). These are then run through the internal generative model to generate samples **y**^(*n*)^. The recognition weights and biases are then adjusted using the same kind of rule (see Equations 5–8 in Methods).

#### 3.1.1. Delta rule network

Each unit in the computational Helmholtz machine is implemented using a microcircuit of spiking neurons (Figure 2A). This network is composed of small pools (10–20 neurons each) making sparse but specific connections (see Table S4 for the connectivity parameters) to each other. The network interacts with other units through an input pool (labeled *E*), an output pool (labeled *O*) and a target pool (labeled *T*). We interpret the mean firing rates of neurons in these pools (averaged across the individual neurons) as the input, output and target activities of the unit this neural network implements. The input and target pools are implemented as generators of Poisson spike trains, while the rest of the neurons in the network follow the dynamics described in Equation (10). On a short time scale, the network implements the unit's conditional distribution (Equations 1, 4); that is, the output rate *r*_*O*_ stochastically samples from the a distribution that is a function of the input rate *r*_*E*_ and internally encoded weights (the encoding is discussed below). On a longer time scale, the network, through synaptic plasticity, adjusts the mean *r*_*O*_ (averaged across that longer timescale) to match *r*_*T*_ in accordance with the delta rule (Equation 22).

These two behaviors are implemented through the use of the remaining pools in the network, labeled *I*, *M*, and *D*. The overall architecture of the network is driven by the constraint that *r*_*O*_ is independent (to maximum extent possible) of *r*_*T*_ on a short timescale (as the conditional distribution the network is implementing has no such short-term dependency) despite *r*_*T*_ making connections into the microcircuit for the purposes of implementing the delta rule (in our model, neurons communicate solely using spikes). This is accomplished by having pool *T* make an excitatory connection onto pools *M* and *O*, while having pool *M* connect to pool *O* through an intermediate inhibitory pool *D*. This arrangement is aided by the approximately linear FI curves of the component neuron types (Figure 2C). The overall effect of this connectivity is that if the net connectivity strength from pool *E* onto pool *M* is excitatory, then increasing *r*_*E*_ will lead to a decrease in *r*_*O*_. The opposite happens if the net connectivity is inhibitory. Thus, this balance between feed-forward excitation and inhibition implements the weights of the computational unit that this network corresponds to.

Pools *I* and *M* are responsible for adjusting that balance in a way consistent with the delta rule. The synaptic strength of connections made by neurons in pool *I* onto pool *M* is governed by a spike-based plasticity rule that implements (under appropriate conditions) a certain form of the rate-based BCM rule (Bienenstock et al., 1982). This rule adjusts the synaptic strength based on the pre-synaptic firing rate (*r*_*I*_) and post-synaptic firing rate (*r*_*M*_):

(23) $$\frac{dw}{dt}=\eta r_{I}r_{M}(r_{M}-r_{\theta })$$

In the classical BCM rule formulation *r*_θ_ would depend on the average of *r*_*M*_ across a long timescale, but in our model it is held constant. The microcircuit implements the delta rule by enforcing the following two constraints. First, when *r*_*T*_ ≥ *r*_*O*_, then that implies that *r*_*M*_ ≥ *r*_θ_. Second, when *r*_*T*_ < *r*_*O*_, then that implies that *r*_*M*_ < *r*_θ_. Given these rate relationship identities, the sign of the weight change that arises from the BCM synaptic plasticity rule (Equation 23) given a certain *r*_*I*_, *r*_*O*_ and *r*_*T*_ matches that arising from the delta rule (Equation 22). The network thus approximates the linear form of the delta rule (Figure 2B, thin line) with the non-linear form of the BCM rule (Figure 2B, thick line). Aside from preserving the sign, this is quite a gross approximation, and verification will be required to see if it still functions correctly in the tasks where it is meant to replace the delta rule. We wish to stress that this approximation will be used well-outside the approximately linear region near *r*_θ_; i.e., we are not linearizing the BCM rule around *r*_θ_. Aside from these plastic connections, the remaining synaptic connections are fixed, having been optimized in order to implement the aforementioned constraints.

#### 3.1.2. Spiking plasticity rules

To fully specify the spiking neuronal network in Figure 2A it is necessary to clarify what is meant by rate (as it has no model independent definition for spiking neurons) and the exact nature of the spike-based synaptic plasticity rule that implements the rate-based BCM rule necessary for the microcircuit's operation described above.

In this paper we will contrast two spike-based rules that both implement the BCM rule for certain classes of pre- and post-synaptic spike trains. The first is a minimal, but unrealistic, implementation that we term the *Spiking BCM* rule. The second is a simplified version of the Triplet Spike-Timing Dependent Plasticity which is already known to be able to implement the BCM rule (Pfister and Gerstner, 2006). We term this reduced version the Spike-Timing Dependent Plasticity of Inhibition, or *STDPi*.

**Figure 3:**
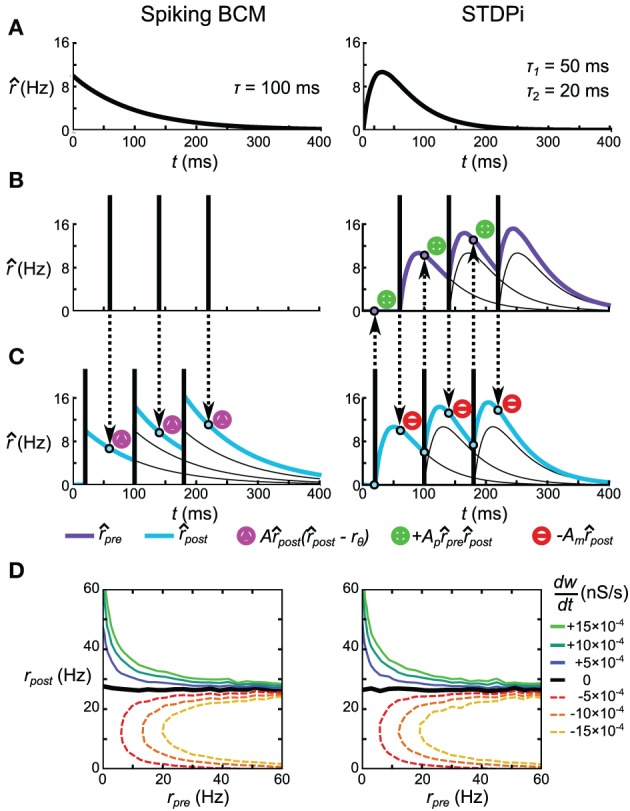
**Spike-based plasticity rules that implement the rate-based BCM rule. (A)** Kernels used to estimate the pre-synaptic (STDPi only) and post-synaptic (both rules) rates. The kernel for Spiking BCM is an exponential kick-and-decay with a single time constant, while the kernel for STDPi is a difference of two exponentials. Both kernels equal zero for *t* < 0. **(B,C)** Schematic diagram of the operation of the two rules given a pre-synaptic spike train **(B)** and a post-synaptic spike train **(C)**. Circumscribed magenta triangles signify when the Spiking BCM adjusts the synaptic weight (both in positive and negative directions). Circumscribed green pluses and red minuses signify when STDPi respectively potentiates and depresses the synaptic weight. **(D)** $\frac{dw}{dt}$ as a function of uncorrelated, Poisson distributed pre- and post-synaptic rates.

The Spiking BCM rule estimates the post-synaptic rate $\hat{r}$_*post*_ by filtering the post-synaptic spike train using an exponentially decaying kernel (Figure 3A, left). At the time of every pre-synaptic spike, the synaptic weight is potentiated or depressed depending on the instantaneous value of $\hat{r}$_*post*_ (Figure 3C, left). The synaptic weight is not adjusted in any way during the post-synaptic spikes.

The STDPi rule filters both the pre-synaptic spike train and the post-synaptic spike train with a difference-of-exponentials kernel (Figure 3A, right) to estimate both the pre- and post-synaptic rates ($\hat{r}$_*pre*_ and $\hat{r}$_*post*_, respectively). During pre-synaptic spikes the weight is depressed in proportion to the instantaneous value of $\hat{r}$_*post*_. During post-synaptic spikes the weight is potentiated in proportion to the product of instantaneous values of $\hat{r}$_*post*_ and $\hat{r}$_*pre*_. This is unlike the Spiking BCM rule where potentiation and depression occur only during pre-synaptic spikes. In this sense, STDPi more accurately resembles the experimental plasticity curves (Haas et al., 2006). This rule is analogous to the simplified triplet-STDP rule explored by Pfister and Gerstner (2006) with the difference being the shape of the kernel (their work used an exponential kernel as we do in the Spiking BCM rule) and the fact that only a single trace is used to estimate the post-synaptic rate (as opposed to two in their work).

If we take two uncorrelated, Poisson distributed pre- and post-synaptic spike trains and run them through these two rules, we observe that they yield identical values for $\frac{dw}{dt}$ as a function of the rates of those spike trains (Figure 3D). This equivalency depends only on the values of *A*, *A*_*m*_ and *A*_*p*_ and τ, but not the time constants of the STDPi kernels. Additionally, it can be seen that the pre-synaptic rate only affects the magnitude of $\frac{dw}{dt}$ and not its sign, just like it does in the BCM rule (Equation 23). In fact, for Poisson distributed spike trains both rules implement Equation 23 exactly. See Supplementary Information for the full derivation of these facts.

#### 3.1.3. Neuronal Helmholtz machine

**Figure 4:**
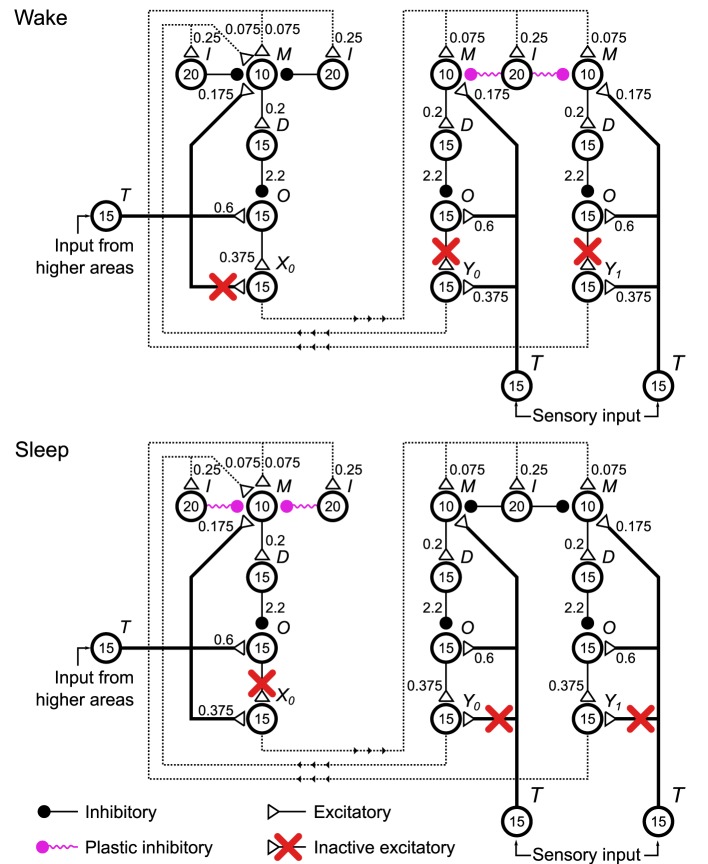
**Configurations of the spiking neuronal network implementation of the Helmholtz Machine during the wake and sleep phases of learning**. Four delta rule networks (Figure 2A), referred to as units within the context of this network, are wired up together to form the Helmholtz Machine, with two handling the sensory layer computation and two handling the hidden layer computation (only one is shown in this figure for clarity). See Figure 2 caption for explanation of the symbols used. To support the two phases of the wake-sleep algorithm some connections (red crosses) are inactivated to control what determines the firing rate of the output pools of each unit (pools *X*_0_, *X*_1_, *Y*_0_ in this figure). In the wake phase (top) the outputs of the sensory units are determined by the sensory input, while the outputs of the hidden units are determined by the firing rates and the corresponding connection strengths of the outputs of the sensory units. In the sleep phase the outputs of the sensory units are determined by the firing rates and the corresponding connection strengths of the outputs of the hidden units, while the outputs of the hidden units are driven by the input from higher areas. Additionally, in accordance with the wake-sleep algorithm, the plastic connection strengths to the sensory units only get modified during the wake phase, and vice versa for the connection strengths to the hidden units. The non-specific external inputs within each unit are not shown. Also not shown are plastic inputs onto the pool *M* of each unit that implement the bias activity and weights. These are modeled as a Poisson spike train with rate *r*_*b*_ = 25 Hz. These plastic inputs form the same type of plastic inhibitory synapse as all the other plastic connections shown on the figure.

To implement the Helmholtz Machine (Figure 1B) we arrange four delta rule networks, or units as we will now call them, (Figure 4, only three units are shown for clarity) into two layers and make connections between the units of different layers. Two of those units correspond to the variable **y** (the sensory layer) and the other two correspond to the variable **x** (the hidden layer). As the delta rule network operates on the firing rates of pools of neurons, the neuronal network implementation of the Helmholtz Machine encodes the values of those variables via rate coding. In this network the mean rates (during a 500 ms window) of pools *X*_0_, *X*_1_, *Y*_0_, and *Y*_1_ (collectively, the output pools) represent the realizations of the random variables *x*_0_, *x*_1_, *y*_0_, and *y*_1_, respectively. The probability distribution over those variables is modeled through the stochastic variability of those rates arising from both the rate variations of non-specific external inputs to the delta rule network pools *M* and *O* (see caption of Figure 2 and Methods) as well as the stochastic synaptic vesicle release within the synapses of the network. By construction, the output pools sample (see Figure S1) from the probability distribution conditioned on the rate of the input pools (i.e., the output pools of other units) weighted by the synaptic strength of the connections they make onto the pool *M* of every unit. Additionally, there are also pools that make plastic connections on the input pools that are active non-specifically. These connections model the biases in the computational model. See Table S4 for connectivity parameters. The computational weights and biases (namely **W**_**G**_, **W**_**R**_, **B**_**G**_, and **B**_**R**_) correspond to the overall effect of the outputs of units on the output rate of a unit that they connect to. In terms of the neuronal network implementation, this corresponds to the balance between feed-forward inhibition and excitation.

The specific external connections are made through the pool *T* of each unit (collectively the external input pools). These are modeled as spike trains with Poisson statistics. In this case, the rate of these processes are held constant for each 500 ms interval, and then a new rate is chosen from the probability distribution in question (the outside world, or the priors).

**Figure 5:**
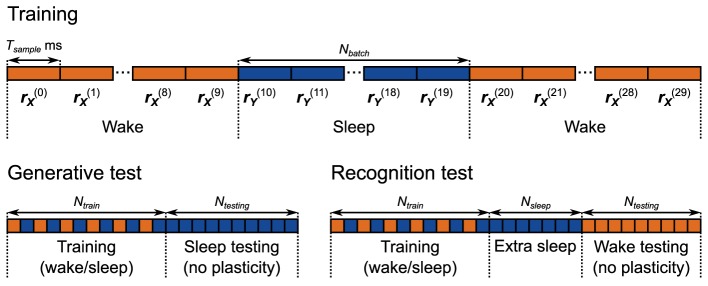
**Training and testing protocols for the neuronal network implementation of the Helmholtz Machine**. The network is trained for 5000 s using alternating wake and sleep phases. Each phase of wake and sleep consists of 10 samples, 500 ms each. During the generative test 2000 samples are collected from the generative model.

The neuronal network uses an adapted wake-sleep algorithm (Figure 5 Training). To support the two phases of the learning algorithm in this network, we introduced two sets of switchable connections. The first set involves connections in each unit that go from pool *T* to the output pool. The second set involves connections in each unit that go from pool *O* to the output pool. These connections control what specifies the realizations of the random variables: the rates *r*_*X*0_, *r*_*X*1_, *r*_*Y*0_, *r*_*Y*1_ in this network. In the computational model, the realization of *y*_0_, for example, can be taken from the environment or from the internal generative model. In sensory units in the neuronal network, for example, this is determined by whether the active connection to the output pool is made from the external input pool, or from the pool *O*. Along the same lines these connections can be thought of as determining whether or not the unit generates a sample from its conditional probability distribution. During the wake phase, for example, to adjust the generative weights we compute **W**_**G**_**x**^(*n*)^ + **B**_**G**_*b* (Equation 6), but we do not then use that reconstructed mean to generate a sample from the internal generative model. In the neuronal network, during the wake phase, for example, the connection between pool *O* of the sensory unit and the output pool is broken, to prevent that sample from being sent out to the hidden units. See Discussion for thoughts about the nature of these switching processes in the brain.

Additionally, the connection strengths to the sensory units (corresponding to the generative weights **W**_**G**_ and biases **B**_**G**_) only get modified during the wake phase, and the connection weights to the hidden units (corresponding to the generative weights **W**_**R**_ and biases **B**_**R**_) are only modified during the sleep phase. The sensory input and the input from higher areas during sleep and wake phases respectively are set to a steady 40 Hz.

Unlike the computational model, each wake and sleep phase consists of multiple consecutive samples per phase. This is done in order to minimize the effect of the rate transients that happen when the network switches between sleep and wake phases. For example when the network switches from the wake phase to the sleep phase, the rate of the *M* pool in the hidden units (Figure 4) switches from operating on samples taken from the environment to operating on samples produced by the generative model. Even though the plasticity is turned off in those units during the wake phase, the kernels that estimate the rate of the neurons in those pools (Figure 3) still function. This means that initially during the first sample of the sleep phase that follows the wake phase, the estimated rate is incorrect, causing errors in learning. A similar issue affects adjacent samples within the wake phase and adjacent samples within the sleep phase, but since the rates are taken from the same distributions (from the environment and from the prior respectively) this is a less severe problem. These issues constrain the temporal scale of the dynamics of neurons, the plasticity rules and the active sensation mechanisms (e.g., saccades). If the environment changes more quickly during the wake phase (or the higher brain regions fluctuate in activity more rapidly during the sleep phase) than the rate estimation mechanism can keep up with, the learning will be adversely affected.

This issue means that the choice of the number of samples per phase can be of critical importance for successful learning. In practice we find that this choice depends on the complexity of the data and prior distributions. Complex tasks (bimodal data sets and priors) require larger batch sizes. We use a batch size of 10 for most tasks, increasing it to 50 for the more complicated recognition tests. Our tests show that once the batch size exceeds a certain amount, the network performance plateaus for small (≤ 50) numbers of samples per phase.

### 3.2. Delta rule network results

**Figure 6:**
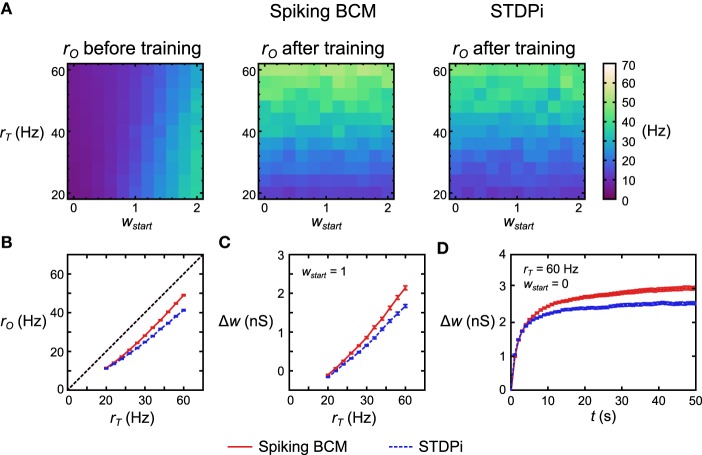
**Performance of the delta rule neuronal network. (A)** For each plasticity rule, 121 structurally identical networks were given different *r*_*T*_ and initial weights *w*_*start*_. The networks were then simulated for 50 s. Before training *r*_*O*_ follows *w*_*start*_ and is largely independent of *r*_*T*_. After training *r*_*O*_ follows *r*_*T*_ (more accurately, it follows *w* which has been trained by *r*_*T*_ as *r*_*O*_ is largely independent of *r*_*T*_ on short time scales). **(B)** Averaging across 50 instantiations of the above 121 delta rule networks and collapsing along the *w*_*start*_ axis, the deviation from *r*_*T*_ is nearly constant for all values of *r*_*T*_ when Spiking BCM is used (red solid), but it is non-constant when STDPi is used (blue dashed). **(C)** Networks that use STDPi have trouble attaining high weights. **(D)** Change in weight over time for one trial of 50 networks. For both plasticity rules the weights approach a certain steady state, but for STDPi this final weight value is lower than it is for Spiking BCM. The error bars are SEM.

To test the functionality of the delta rule neuronal network, we took 121 separate but structurally identical networks for each plasticity rule. Each network received a different target rate *r*_*T*_ and different initial mean weight of the plastic connections *w*_*start*_. All other parameters were kept the same, with *r*_*E*_ being set at 20 Hz. We then simulated each network in these conditions and recorded the *r*_*O*_. Before training, *r*_*O*_ increased with increasing *w*_*start*_ (Figure 6A, left). The variation in *r*_*O*_ as a function of *r*_*T*_ before training comes from the imperfect linearity of the network. After 50 s of training, *r*_*O*_ now follows *r*_*T*_ when the network used the Spiking BCM rule and when it used STDPi (Figure 6A, middle, right). Note that if we change *r*_*T*_ on the short time scale, *r*_*O*_ will remain unaffected (modulo the imperfections mentioned above): the pattern of variation in *r*_*O*_ comes from *r*_*I*_ affecting it differently based on the trained weights. To examine more clearly how well *r*_*O*_ matches the training *r*_*T*_ we simulate 50 instantiations of the random connectivity for each of the 121 networks above and average across the starting weights. For the Spiking BCM rule we note that the deviation between *r*_*T*_ and *r*_*O*_ is approximately constant for all *r*_*T*_ (Figure 6B, red line) while for STDPi this deviation increases for higher *r*_*T*_ (Figure 6B, blue dashed line). If we look at the changes in weight given *w*_*start*_ = 1 (Figure 6C), it can be seen that this is because the STDPi does not increase the synaptic weight quite as much as Spiking BCM does in those conditions. This is not an issue of convergence, as looking at the total weight change over time for one trial averaged across 50 networks it is clear that both weights converge for both rules during the training (Figure 6D). Despite these imperfections, at least in this simple task, the use of the approximate delta rule and the implementation of it using the spiking plasticity rules does not lead to catastrophic degradation of performance and we can move on to try using this network as part of the Helmholtz Machine.

### 3.3. Generative model results

#### 3.3.1. Training protocol

First we test the computational model and the neuronal network in the generative mode. That is, we examine how well the model matches the probability distribution over the input units on which it was trained. This is most applicable to matching spontaneous *in vivo* neural activity in the early cortical areas (e.g., early sensory cortices Berkes et al., 2011). The computational model and the neuronal network are trained on nine data sets (labeled *a* through *i*). Data sets *a* through *e* consist of skewed and unskewed unimodal bivariate truncated gaussians, while data sets *f* through *i* are bimodal mixtures of unskewed bivariate gaussians. For the unimodal data sets we use a unimodal prior distribution (Equation 15) while for bimodal data sets we use a bimodal prior distribution (Equation 16). In the neuronal networks, the different prior distributions are implemented by altering the firing rate distribution of the pool *T* in the hidden units during the sleep phase. Such a manual task-dependent choice of priors is necessary because our model contains only a single hidden layer; the power of the Helmholtz Machine is in its ability to be implemented in a hierarchical structure, so that the type of prior would be learned as the connection strengths for the second hidden layer in a larger model.

The models are trained and tested following the protocol depicted in Figure 5, Generative Test. Each session starts with a training period with a total duration of 5000 s (for a total 500 wake phases and 500 sleep phases, where each phase comprises 10 samples) for the neuronal network. The computational model is trained for 250,000 wake and sleep phases (with one sample per phase). We use a relatively faster learning rate for the neuronal network for the sake of computational efficiency. At the same time, however, it is 90% slower than it was in the delta rule network because the weights do not converge correctly if the learning rate is too fast. This was not an issue in the delta rule network due to the steady inputs it received during training. The presentation order of the data was random for each session (see Materials and Methods). Each session, after training, we examine the generative model of both the neuronal network and the computational model by collecting 2000 samples from it. We examine 50 separate networks, each having a different instantiation of the random connectivity, in order to examine the effect of the connectivity issues discussed above. We record one such session per network.

#### 3.3.2. Unimodal training data sets

**Figure 7:**
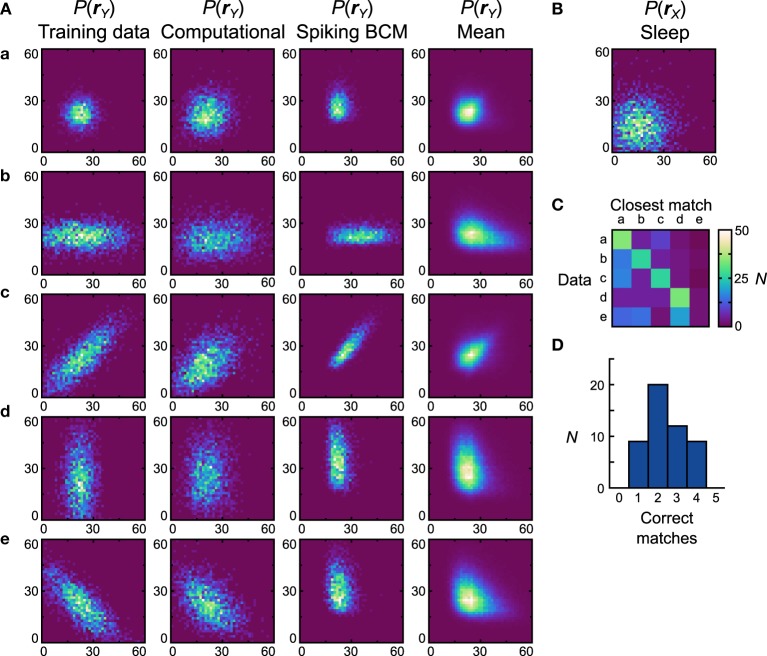
**Performance of the neuronal network implementation of the Helmholtz Machine with the Spiking BCM rule and unimodal data sets. (A)** The computational model and 50 instantiations of the neuronal networks were trained on five unimodal data sets shown in the first column. The axes range from 0 to 60 Hz on both components of **r**_**Y**_. The resultant generative models from the computational model are shown in the second column. The generative models of the best performing (in terms of matching the training data distributions with the learned generative models) neuronal network are shown in the third column. The fourth column depicts the population means of the generative models of the neuronal networks. **(B)** Prior distribution used for the generative models. The axes range from 0 to 60 Hz on both components of **r**_**X**_. **(C)** Confusion matrix obtained by matching the generative models of individual networks with the average (computed across all networks for a given data set) generative distributions. **(D)** Histogram of the number of correct matches by each neuronal network.

We first examine how the neuronal networks perform when trained on unimodal data sets (Figure 7A, first column) with a unimodal prior (Figure 7B). To have a point of comparison, we also examine the histograms of the generative models obtained from the computational model trained on the same data sets and with the same prior (Figure 7A, second column). Despite using an approximate learning rule (Equation 9) the computational model manages to accurately learn the generative models. We quantify this by computing the similarity between the generative models and all the training data sets. We measure similarity using the Jensen-Shannon divergence (Lin, 1991), which ranges from 0 for perfectly identical probability distributions to 1 for distributions with no overlap. We look at *D*_*net*_ which is the average divergence across the data sets, and *D*_*pop*_ which is the average *D*_*net*_ across all random instantiations of the model.

Once we compute the similarity matrix we look at the data set that is most similar to the generative model and, if it matches the data set that was used for training, we state that the model has correctly learned the data set. By this metric the computational model learned all the presented data sets (*D*_*net*_ = *D*_*pop*_ = 0.19). For the neuronal network implementation we examine the two plasticity rules separately. We generate 50 separate networks, each with a different instantiation of random connectivity and examine performance across these network realizations. Starting with networks that used the Spiking BCM rule, the generative models of the best network (i.e., of the one that made the most matches) are plotted in Figure 7A, third column (*D*_*net*_ = 0.35). In all instantiations, the neural implementation failed to match the data set *e* (*D*_*pop*_ = 0.39 ± 0.012 SEM).

Data set *e* presents a challenge to the neuronal network because it requires the components of **y** to be anti-correlated, something that in this model can only be achieved with negative generative weights. The delta rule network is capable of representing negative weights by adjusting the balance between the excitatory and inhibitory feed-forward input connections. The range of weights that it can represent is not symmetric about zero, however, making it impossible to produce the very negative weights required to model some distributions. The reason for such asymmetry stems from the fact that only inhibitory connections are plastic in our network. A strong negative weight requires strong feed-forward excitation, which is difficult to counteract with plastic inhibition when non-negative weights are required. Therefore, a relatively weak feed-forward excitation is used, which leads to a limited ability to represent negative weights. This issue can be resolved through the use of a more sophisticated population coding method (see Discussion). Thus, we do not foresee this to be an actual problem in the brain.

Since the networks produced quite different generative models from the data sets they were trained on, it is more informative to compute the similarity matrix with respect to the probability distributions obtained by averaging the generative models of all networks trained on a particular data set (Figure 7A, forth column). This analysis will show whether the generative models learned by the networks are different for different data sets. If the networks do this task perfectly, then the data set of the average distribution that a network's generative model is most similar to will match the data set the network was trained on. We can plot this using a confusion matrix (Figure 7C) for all of the neural networks. We can see that for the first four data sets most networks produce generative models that match the average distribution well, but fail when trying to match the average distribution of the data set *e*. A performance histogram showing the number of correct matches per network (Figure 7D) reveals that no network matches all five data sets, with most networks matching only two.

Next we examine how neural Helmholtz machines with the STDPi plasticity rule perform on the same unimodal data sets. When this rule was used in the isolated delta rule networks, deviations in performance from the Spiking BCM rule could already be seen, so we also expected differing performance in this task. One of the results from the investigation of the delta rule network was that when it used the STDPi rule, it could not match high target rates (Figure 6B). To compensate for this, we multiplied by three all of the prior rates used in both the computational model and the network with the spiking BCM plasticity rule (the training data sets were kept the same). An unfortunate side effect of this is that the detailed behavior of the network obtained using this rule can only be compared in a qualitative way to that produced by the Spiking BCM rule.

**Figure 8:**
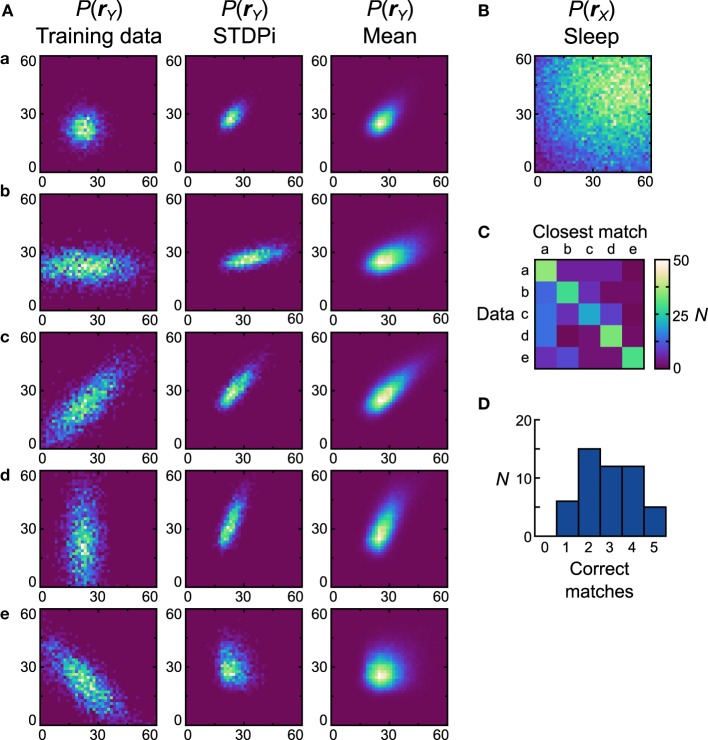
**Performance of the neuronal network implementation of the Helmholtz Machine with the STDPi rule and unimodal data sets**. See Figure 7 for explanation of the panels. The parameters for the STDPi kernels are, τ_1_ = 50 ms, τ_2_ = 20 ms.

Figure 8A shows the data sets (first column), the generative models of the best network (second column) and the average distributions (third column). The prior used is shown in Figure 8B. *D*_*net*_ of the best network is 0.47 and *D*_*pop*_ = 0.53 ± 0.13 SEM. It is clear that networks that use the STDPi have trouble matching the data even qualitatively (the best network only matches the first three data sets correctly). The networks tend to produce positively correlated probability distributions regardless of the training data, although the level of correlation is modulated in the correct direction. As before, we also examine how distinct are the generative models that are trained on different data sets by computing a confusion matrix (Figure 8C) and a corresponding match performance histogram (Figure 8D). Despite the relatively poor performance in matching the data distributions, the networks do learn distributions that are different when trained on different data sets. Five (10%) networks matched all five data sets, although, as with the Spiking BCM rule, most matched only two.

#### 3.3.3. Bimodal training data sets

**Figure 9:**
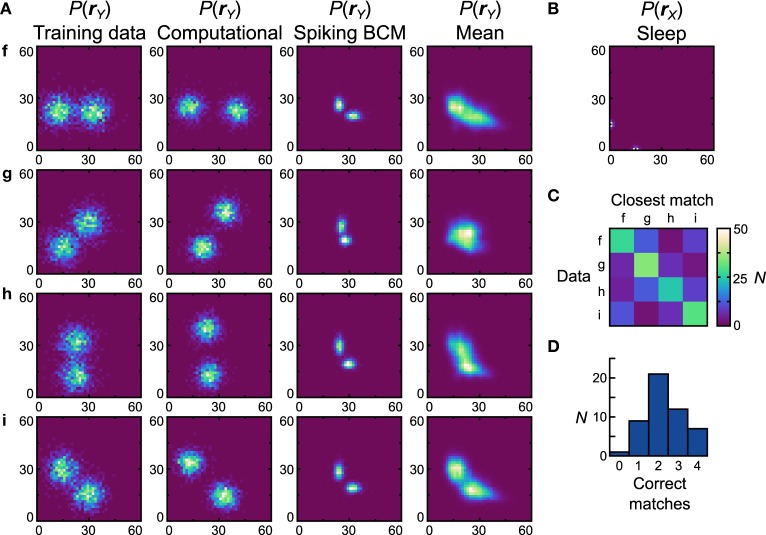
**Performance of the neuronal network implementation of the Helmholtz Machine with the Spiking BCM rule and bimodal data sets**. See Figure 7 for explanation of the panels.

Figures 7, 8 is performed with the bimodal data sets (Figure 9A, first column) and prior (Figure 9B). Again, the computational model has no trouble with these data sets (Figure 9A, second column), leading to a close qualitative and quantitative match (perfect match performance when using the match test, *D*_*net*_ = *D*_*pop*_ = 0.30). Starting with the Spiking BCM plasticity rule, the generative models from the best performing neuronal network (Figure 9A, third column) resemble qualitatively the data sets they were trained on, and while the matching performance is perfect, the *D*_*net*_ is a relatively poor 0.62 (*D*_*pop*_ = 0.58 ± 0.0075 SEM). Examining the average distributions it is clear that most networks do not perform well with data set *g*. The reasons for this are similar to the reasons the neuronal networks perform poorly with data set *e*, namely the need for strong negative connections. Doing the matching test on the average distributions yields a confusion matrix (Figure 9C) and a performance histogram (Figure 9D). Seven (14%) networks match all four average distributions.

**Figure 10:**
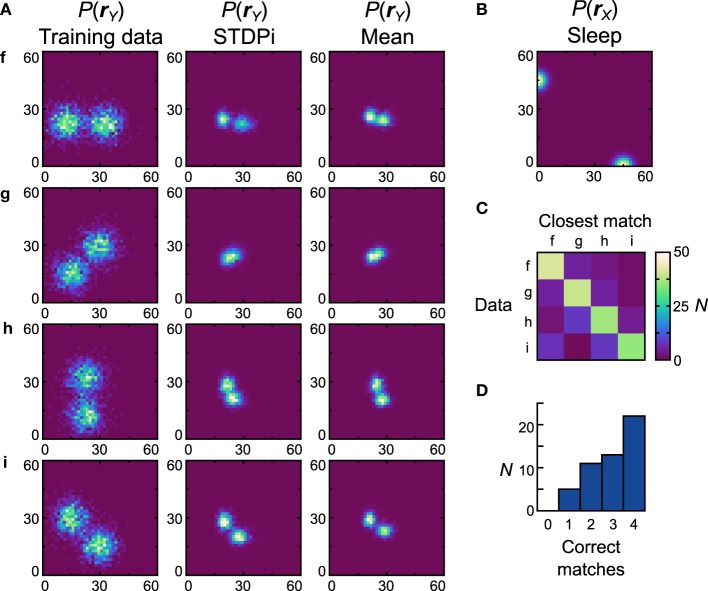
**Performance of the neuronal network implementation of the Helmholtz Machine with the STDPi rule and bimodal data sets**. See Figure 7 for explanation of the panels. The parameters for the STDPi kernels are, τ_1_ = 50 ms, τ_2_ = 20 ms.

The networks using the STDPi rule fare better with the bimodal data sets (Figure 10A, first column) and a bimodal prior (Figure 10B). The generative models of the best network look qualitatively similar to the data sets (Figure 10A), second column) although the *D*_*net*_ is a high 0.53 (*D*_*pop*_ = 0.59 ± 0.0082 SEM). One exception is the data set *g*, which is difficult to learn as it relies on a good representation of negative weights. Looking at the confusion matrix (Figure 10C) and the match performance histogram (Figure 10D) we see how remarkably different the learned distributions stemming from training on different data sets are. Twenty-two (44%) of the networks match all the average distributions (Figure 10A, third column) correctly.

Overall, it appears that going from the computational model to the neural implementation affects the performance in non-trivial ways. Despite both the neuronal network implementation and the computational model using an approximate learning rule, the neuronal networks do quantitatively worse by every metric shown here. This reduced performance is a combination of the imperfections already shown in the delta rule network results (Figure 6) combined with the previously mentioned effects of cross-talk between learning phases and the imperfection of the switching connectivity. Additionally, fundamental issues of weight representation affect some classes of data distributions and not others.

#### 3.3.4. The effect of pre- and post-synaptic spike correlations on the STDPi rule

STDPi and BCM plasticity rules are identical for certain classes of pre- and post-synaptic spike trains and, when used in a more complicated and realistic environment of the delta rule network, they also show relatively small quantitative differences. In the more sophisticated setting of the neural Helmholtz Machine, however, these minor quantitative differences are amplified into qualitative effects. The STDPi rule does relatively poorly when networks that use it are required to learn a probability distribution, and even provision of a more favorable prior distribution does not resolve all of the issues.

**Figure 11:**
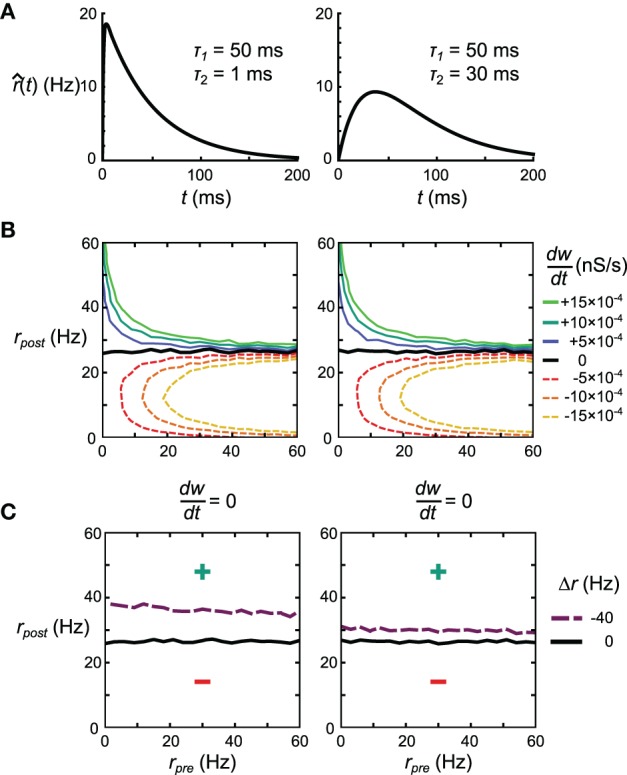
**Effect of kernel shape on the behavior of the STDPi plasticity rule. (A)** The two kernel shapes that are considered in this figure. The same kernel is used for both pre- and post-synaptic rate estimation. **(B)** $\frac{dw}{dt}$ as a function of uncorrelated, Poisson distributed pre- and post-synaptic rates for the kernel with τ_2_ = 1 ms (left) and τ_2_ = 30 ms (right). **(C)** The $\frac{dw}{dt}$ = 0 nullcline for uncorrelated Poisson distributed pre- and post-synaptic spike trains (black) and negatively correlated, Poisson distributed pre-synaptic and inhomogeneous-Poisson post-synaptic spike trains (purple). The kernels used have τ_2_ = 1 ms (left) and τ_2_ = 30 ms (right). All other contour lines have been omitted for clarity.

The explanation for the discrepancy in the apparent similarity between Spiking BCM and STDPi rules shown in Figure 3C and their dissimilarity in performance in the delta rule network and the Helmholtz Machine lies in the short-term correlations between pre- and post-synaptic spike trains due to inhibitory synapses (the relevant excitatory synapses are relatively weak in this model). It is possible to make the kernels used to estimate the rates less sensitive to these correlations by decreasing the contribution of the parts of the kernels most affected by those correlations. Specifically, we adjust the kernel shape such that the interval just after the pre-synaptic spike contributes less to the rate estimate. We do this by altering τ_2_, which governs the width of the initial dip of the STDPi kernel (Figure 3A). To show the general effect this parameter has on the behavior of the rule we examine two extreme values of τ_2_, 1 ms, and 30 ms. The kernels for these values of τ_2_ are shown in Figure 11A. For symmetry we use the same kernel to estimate both the pre- and post-synaptic rates, although the post-synaptic kernel shape is largely irrelevant for this analysis. First, we verify that STDPi using both kernels produces the same behavior as shown by the original STDPi kernel (with τ_2_ = 20 ms) as shown in Figure 3B when the spike trains are uncorrelated. Figure 11B shows that as we vary the pre- and post-synaptic rate we get the same behavior across the two kernel shapes. Next, we generate spike trains with short-term negative correlations. The spikes are generated from the rate expressions using a Poisson process (homogeneous in the pre-synaptic case and inhomogeneous in the post-synaptic case, see Materials and Methods). When we apply STDPi using the two different kernels on such correlated spike trains, we find, as expected, that the correlations do indeed alter the net synaptic plasticity as a function of mean pre- and post-synaptic rates. In particular, the biggest change is the increase in the post-synaptic rate for which the synaptic weight is stationary over time (Figure 11C, black line—uncorrelated, purple dashed line—correlated). This happens because the pre-synaptic rate is underestimated, which causes the synaptic weights to get less potentiated. Importantly, we see the benefit of the larger τ_2_ (Figure 11C, right panel), which ameliorates not only the net underestimation of the pre-synaptic rate—and hence the mean shift in post-synaptic rate at which synapses no longer change strength—but also the dependence of the location of the nullcline on the pre-synaptic rate. The latter is important as to reproduce a perfect delta rule, the steady state post-synaptic rate should be the only factor affecting the direction of change of this synapse.

As described previously, results produced by these artificial examples do not necessarily predict performance in actual networks. Therefore, we test how both the delta rule network and the neural implementation of the Helmholtz Machine depend on the choice of τ_2_.

**Figure 12:**
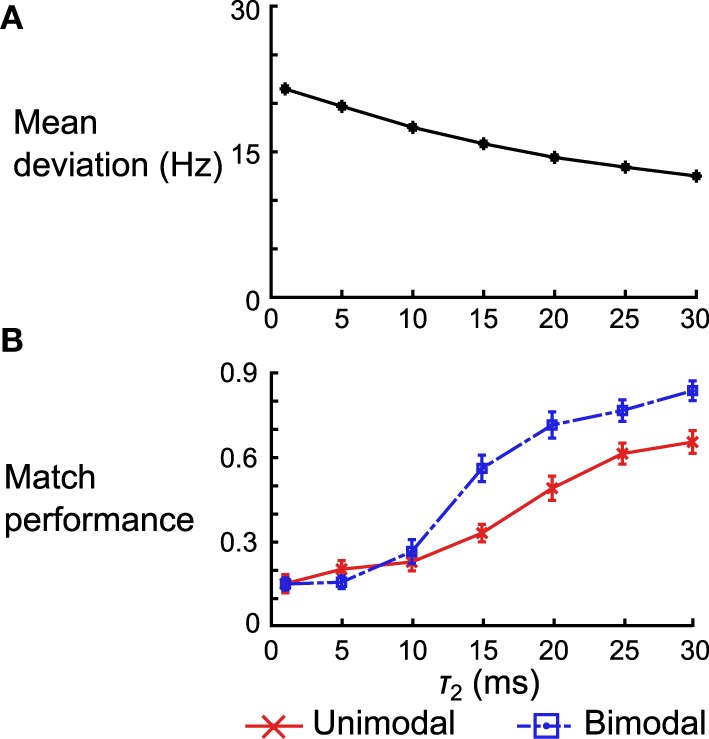
**Effect of kernel shape on the performance of the delta rule network and the neural implementation of the Helmholtz Machine. (A)** Mean deviation (across 50 instantiations of the network) between the output rate after training and the target rate across 50 instantiations of the delta rule network. The error bars are SEM, but are too small to be seen in this plot. **(B)** Mean matching performance for unimodal and bimodal data sets across 50 instantiations of the neural implementations of Helmholtz Machine. The error bars are SEM. Note that the performance is normalized by the number of data sets (e.g., a performance of 1.0 means all 5 mean distributions were matched in the unimodal data set category).

For the delta rule network we quantify the performance by looking at the average deviation of the output rate after training from the target rate. We vary τ_2_ and look at the mean deviation across 50 networks (differentiated by the instantiations of the random connectivity). We see that the deviation decreases with the increasing τ_2_ (Figure 12A). When τ_2_ = 30 ms the mean deviation across different networks is 12.50 ± 0.26 (SEM) Hz, which is just shy of the mean deviation of 10.98 ± 0.25 (SEM) Hz obtained when using the Spiking BCM rule.

For the neural implementation of the Helmholtz Machine we focus on the matching test performed in panel D of Figures 8, 10. We look at both the unimodal and bimodal data sets and we normalize the network performance by the number of data sets in that group (i.e., instead of ranging from 0 to 5 for the unimodal data sets, it now ranges from 0 to 1). We examine performance across 50 instantiations of the Helmholtz Machine neuronal networks while varying τ_2_ as before. While match performance increases for both data sets when τ_2_ is increased, it increases much more dramatically for the bimodal data sets (going from matching an average of 1.26 data sets to 3.40 data sets). This is, in part, because the networks that use poor kernels tend to not learn weights that produce bimodal generative models. As soon as the kernels get good enough (τ_2_ > 10 ms) to separate the two modes, performance increases dramatically.

### 3.4. Recognition model results

So far we only tested our Helmholtz Machine implementation in the generative mode. During behavior and perception, however, the animals will likely use the recognition model to perform inference. Thus, we will now explore how well the neuronal networks function in recognition mode. This mode is most applicable to matching neural data in the higher cortices, as well as behavioral data. We focus on behavioral tasks in this section as the behavioral data is more readily obtainable.

#### 3.4.1. Linear decoding and sleep improvement

**Figure 13:**
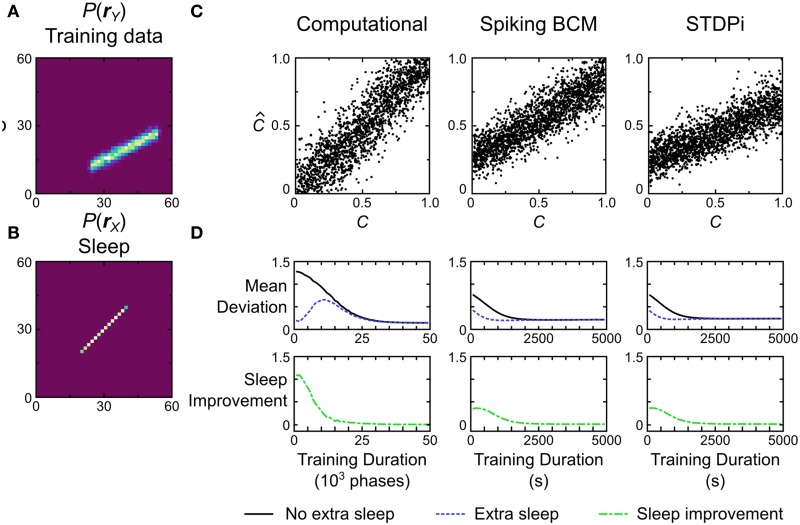
**Performance of the computational and neuronal network models on a decoding task. (A)** Data set *o* that the models are trained on for this task. Each data point is labeled with a label *C* signifying its position on the line. The position ranges along [0, 1]. The models have to recover this position given only the data. **(B)** Prior distribution used during training. **(C)** Decoding performance of three example model instances. *C* is the true value used to generate a data point, while $\hat{C}$ is the value that the model estimated from the data. **(D)** If the training is stopped before the weights have converged, additional samples in the sleep phase without further data presentation can lead to improvement in performance. Error bars have been omitted for clarity.

The first test we perform is a simple linear decoding test. The models are trained using a uniform line data set (Figure 13A, data set *o*), and prior (Figure 13B). During testing, the models have to determine where a presented data point is on the line (position in this case is a 1-dimensional quality ranging from −1 to 1). This is achieved by looking at where the activity of the hidden units lies on the prior (which is also the line). The critical point here is that the decoding strategy used to score the model is explicitly specified by the prior distribution, which means that the transformation from the data distribution to the distribution of decoded positions is entirely learned by the model (see Materials and Methods for details of the decoding procedure). The models are trained and tested following the protocol depicted in Figure 5, Recognition Test. During each session the computational model is trained for 50,000 phases (25,000 wake and 25,000 sleep phases) while the neuronal networks were trained for 5000 s (500 wake and 500 sleep phases with 10 samples per phase). To examine the performance of the computational model, it is simulated 50 times (with separate instantiations of the temporal stochasticity). For the neuronal network we generate 50 networks with different instantiations of the random connectivity per plasticity rule. Each network is tested using one session as described above.

Figure 13C shows the performance of the computational model and two instantiations of the neuronal network models, one using the Spiking BCM plasticity rule and one using the STDPi plasticity rule. The computational model performs the decoding without any appreciable bias, while the neuronal networks show bias and inability to decode the entire dynamic range of the data. We can quantify the performance by computing the mean deviation (defined as the square root of the mean squared error, averaged across trials) between the true positions of the data points and the decoded positions. The computational model does the best at 0.15. The neuronal networks using the Spiking BCM rule do worse with a mean deviation of 0.22. Networks with the STDPi rule do a little worse still at 0.24.

This decoding test is a good way to illustrate a prediction arising from the means by which any implementation of the Helmholtz Machine learns the recognition weights. Since the task performance solely depends on recognition weights, and the recognition weights are learned during the sleep phase, we may observe improvement in performance across a sleep phase without presenting any additional training data. This is trivially true for the short sleep phases used during training, because without any improvement we would not observe the overall improvement in performance throughout the entirety of the wake-sleep training. However, this is not obviously true for longer sleep phases with many more samples. Early on in the training prolonged sleep would produce a converged recognition model that inverts an incompletely converged generative model, and there is no guarantee that this recognition model is any better than an unconverged recognition model of the same generative model.

To examine this effect we first track the mean deviation for the models during training (Figure 13D, black curve). The initial weights for all models were chosen to be small so the initial mean deviation is correspondingly large. We use relatively slower learning rates for all models to visualize the improvement in performance over time, as this test is very easy and the models would learn it too quickly otherwise. At certain intervals we stop the presentation of data, and repeatedly sample in the sleep phase until the recognition weights converge. Examining the mean deviation of the models (Figure 13D, blue curve) shows that there is a distinct reduction in mean deviation brought about by sleep, without any new presentation of data. The green curve in Figure 13D shows the magnitude of this improvement at various times during the training. Not surprisingly the biggest improvement is produced early in the training, while late in the training little is gained by such prolonged sleep phases.

#### 3.4.2. Biased reward

**Figure 14:**
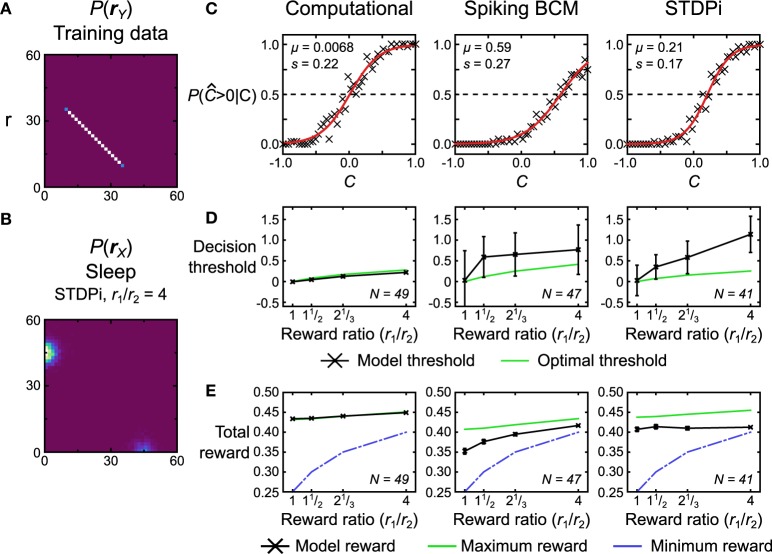
**Performance of the computational and neuronal network models on a reward task. (A)** Data set *r* that the models are trained on for this task. Each data point is labeled with a label *C* signifying its position on the line. The position ranges along [−1, 1]. The models have to determine whether this position is positive or not, with the two alternative choices being rewarded by different amounts. **(B)** The bimodal prior for this task encodes the reward ratio *r*_1_/*r*_2_ of the two alternative choices by modulating the weightings of the two gaussian blobs. The gaussian blob positions and sizes are the same as in Figures 9, 10. Depicted is the prior used for the neuronal networks using the STDPi plasticity rule when *r*_1_/*r*_2_ = 4. **(C)** Response probability of three example model instances for *r*_1_/*r*_2_ = 1. The probability data (crosses) is fit with a logistic function (red line) to determine the model's internal noise and decision threshold. Note that the two neuronal networks show a decision threshold bias. **(D)** Model decision threshold (black) compared with the optimal threshold (green) given the model noise level (estimated from trials where *r*_1_/*r*_2_ = 1). Error bars are standard deviation. *N* is the number of models that were used for a plot. **(E)** Model attained reward (black) compared with the maximum that an optimal decoder with matched noise level would get (green) and the minimum possible given the scoring procedure (blue). A noiseless optimal decoder would attain a reward of 0.5 across all conditions. Error bars are SEM. *N* is the number of models that were used for the plotted data points.

**Figure 15:**
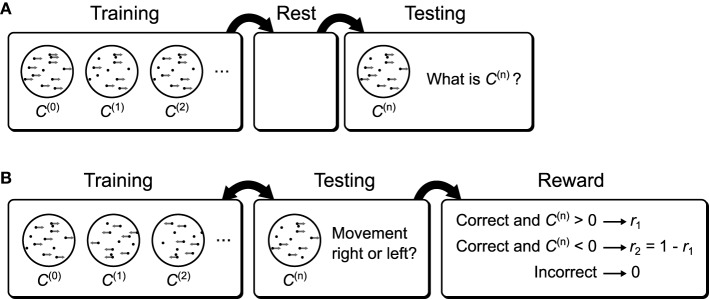
**Possible behavioral tasks that implement the recognition tests. (A)** A possible behavioral task to test the sleep improvement prediction. Subjects are first shown moving dot displays of varying coherence. After training the subjects are allowed to rest. During subsequent testing the subjects are again shown moving dot displays of varying coherence, but now they are asked to report the coherence. If the brain uses a Helmholtz Machine to learn recognition weights then rest will result in improved performance relative to a lack of a rest period between training and testing (Figure 13D). **(B)** A possible behavioral task to examine the effect of reward bias on decision thresholds. Subjects are shown moving dots displays of varying coherence and one of two directions. At the same time they are asked which direction they perceive the dots to be moving and are rewarded differentially based on the true direction of movement. If the brain uses a Helmholtz Machine to learn the recognition weights for this task then the subject's decision thresholds will be over-shifted relative to the optimal thresholds.

The second test we perform is a two-alternative forced choice task with unequal rewards for the two choices. We use a training data set that is a uniform line data set (Figure 14A, data set *r*) and a bimodal prior (e.g., Figure 14B shows a distribution used for neuronal networks that use the STDPi rule). We call the position along the data line *C* (for coherence; see Discussion and Figure 15 for a behavioral task interpretation of this test) ranging from −1 to 1. During testing, the models have to determine whether *C* is less than or greater than 0. This is achieved by noting which of the two hidden units has greater activity (i.e., whether *y*_1_ ≥ *y*_2_ or *y*_1_ < *y*_2_). The models thus can only report if *C* was greater than or less than 0. When the model indicates correctly that *C* > 0, it gets rewarded with a reward value of *r*_1_, whereas if it indicates correctly that *C* < 0, it gets rewarded with a different reward value of *r*_2_. The two reward values are constrained to sum to 1, so a perfect, noiseless decoder applied to noiseless data (as it is in this case) would receive a total reward of 0.5 on average. The reward does not impact the plasticity rules—the Helmholtz Machine uses an unsupervised learning algorithm—but it does affect the prior distribution (in the brain the prior distribution, in this case, would be modified by reward-dependent plasticity). The two truncated gaussians that are mixed to produce the prior distribution are mixed in proportion *r*_1_/*r*_2_ (Figure 14B shows the prior distribution with *r*_1_/*r*_2_ = 4). The activity of the hidden units, therefore, represents the reward levels associated with the observed unit activities. This task is difficult for the neuronal networks when the reward ratio is high (i.e., one mode is a lot larger than the other), so we train the networks for longer periods of time (7500 s) and use larger batches of samples (50) in each wake and sleep phase. As before, we simulate the computational model 50 times. For the neuronal network we generate 50 networks with different instantiations of the random connectivity and then test each one on the whole training data set.

We first quantify the performance of the models by examining how the response probability [*P*($\hat{C}$ > 0|*C*)] depends on the true value of *C*. Figure 14C shows this data for a single run of the computational model and two realizations of the neuronal networks, one using the Spiking BCM rule and another using the STDPi rule. For these three plots the reward ratio is set at unity. We fit the data with a logistic function that is parameterized by its location μ, which we term the decision threshold, and its scale *s* which measures the internal noise of the system:

$$P(\hat{C}>0|C)=\frac{1}{1+\exp\left(-\frac{C-\mu }{s}\right)}.$$

The first observation is that both neural and computational models are noisy and therefore cannot reach the theoretical maximal reward of a noiseless decoder. This also means that the decision threshold will vary with the reward ratio: an optimal noisy decoder will bias the decision threshold away from the choice that has the greater reward (see Materials and Methods for a derivation of this fact). Another observation is that the decision thresholds of the neuronal networks are not zero even when the rewards for each of the two choices are equal (the optimal threshold is 0 in this case). This is caused by the random connectivity within the neuronal networks. We plot how this decision threshold varies as a function of reward ratio (that is, the prior distribution which reflects this ratio) and compare it to the optimal shift given the noise within the model (Figure 14D). The noise is estimated from trials with reward ratio set at unity. The computational model adjusts its threshold nearly optimally, but the neuronal networks over-shift the threshold significantly, i.e., the more highly rewarded choice is selected even more often than is optimal. This phenomenon has been observed in experiments with monkeys (Feng et al., 2009). We also look at the actual average total reward the models obtain (Figure 14E, black curves). We can compare the obtained reward to the theoretical minimum and maximum rewards (green and blue curves on Figure 14E, respectively). The maximum reward is obtained using the optimal threshold placement and thus is model specific. The minimum reward is obtained if we select the threshold so that the model always responds with the most rewarded choice. The computational model essentially gets the maximum reward possible given its internal noise level. Neuronal networks do not perform as well. The poor performance of the neuronal networks in part stems from the fact that the extremely simplistic decoder we use to extract decisions from the neuronal networks does not take into account the bias arising from the random connectivity. These results, therefore, represent the lower bound on performance. This lower bound could be improved with some simple modifications to the decoder (adding homeostatic synaptic scaling (Turrigiano and Nelson, 2004), for example).

## 4. Discussion

We have presented a network of spiking neurons that implements the Helmholtz Machine and its associated unsupervised learning algorithm, the wake-sleep algorithm. In order to produce such a model, we also developed a smaller circuit that implements the delta rule, an error-correcting rule that underlies the learning in the wake-sleep algorithm. We have shown that this model can learn a generative model that models the probability distributions of data sets that the network was trained on. Additionally, we have shown that it can perform approximate probabilistic inference in two recognition tasks. Throughout this work we have contrasted two synaptic plasticity rules, Spiking BCM and STDPi, as putative mechanisms to implement the delta rule and produce the required learning in the Helmholtz Machine. While STDPi is based on biological observation, it leads to performance that matches that of the less biologically constrained Spiking BCM rule in many, but not all, tasks.

The generative tests that we have performed can be used to explain data that shows similarity between stimulus-evoked neural activity and spontaneous neural activity (Han et al., 2008; Berkes et al., 2011; Okun et al., 2012), as well as providing a normative explanation for the sleep replay of neural activity (Sutherland and McNaughton, 2000). The two recognition tests can be applied to behavioral experiments in which subjects have to observe a stimulus and then make decisions based on their inferred percept. Figure 15 shows two possible experiments which utilize moving dot displays that would address the results of the recognition tests. Figure 15A shows a decoding experiment which would explore the effect of rest (additional post-training samples in the sleep phase) on decoding performance. Figure 15B shows a biased reward experiment that would explore the suboptimal decision threshold shifts predicted by our model. Notably, experiments (with a slightly different task) that show suboptimal shifts of decision thresholds in monkeys already exist (Feng et al., 2009).

The neuronal network we propose is not the first that implements the delta rule, although to the best of our knowledge it is the first that fulfills the requirements brought about by our neuronal network implementation (using spiking neurons with rate as a continuous variable and avoiding temporal acausality) of the Helmholtz Machine. By carefully controlling the post-synaptic activity of a synaptic connection, the strength of which is otherwise adjusted by a BCM-like rule, Hancock et al. (1991) implemented a partial delta rule for binary units. This implementation is inadequate for this Helmholtz Machine because it uses continuous valued units. More recently, by combining spike frequency adaptation and spike-timing dependent plasticity (D'Souza et al., 2010) implemented the delta rule for temporally separated, but otherwise continuous units. The nature of the temporal separation requires the target activity to appear after the network's activity. Such a temporal separation would require a breaking of causality in any neural implementation of the Helmholtz Machine because in reality target activity appears before the activity produced by the network. For example, during the wake phase, the target activity is set up by the stimulus while the network's current activity arises from the top down connections that are excited by the stimulus. It is possible that delay lines or post-inhibitory rebound spiking could produce the necessary ordering of activity, but we chose to follow an approach that did not require such additional complications.

As part of the choice of implementing the Helmholtz Machine, we also simultaneously chose to represent probability distributions using samples. This approach has been previously explored by others (Fiser et al., 2010; Buesing et al., 2011; Pecevski et al., 2011; Nessler et al., 2013) and has multiple interesting theoretical properties (see Fiser et al., 2010; Lochmann and Deneve, 2011 for a review), as well as being directly amenable to implementing biologically plausible learning algorithms (Nessler et al., 2013 and this work). A popular alternative represents the probability distributions using probabilistic population codes (Rao, 2005; Ma et al., 2006, 2008). This framework has broad experimental support, although no biologically plausible learning algorithm has been proposed in this framework yet. Yet another alternative for encoding a probability distribution would be predictive spike coding (Deneve, 2008a,b), which supports both inference and learning on a level of individual neurons, but does not extend naturally to representing continuous variables.

The Helmholtz Machine implemented in this paper is very simple, consisting of only one input layer and one hidden layer with two linear units each. As a result, many of the functions achieved by this particular instantiation and presented in this paper can be performed by simpler models without the need for complicated circuits and the wake-sleep algorithm. The power of the Helmholtz Machine, however, lies in its ability to be extended with multiple layers and multiple units, as well as with different conditional probability distributions (Hinton and Dayan, 1996). We chose to restrict ourselves to a very impoverished model to clarify how and why its performance is affected by the neural implementation. Additionally, there are more powerful extensions of the Helmholtz Machine with intra-layer connectivity (Hinton and Dayan, 1996; Dayan, 1999) which still utilize the wake-sleep algorithm. Future implementation of these ideas will allow the proposed connectivity of the neuronal networks to be more consistent with the available neurophysiological data.

Connections between layers in our network are implemented using feed-forward excitatory and inhibitory synapses, of which only the inhibitory ones are plastic. This, however, is not an essential requirement. The same functionality can be implemented even if both types of synapses are plastic or only the excitatory synapses are plastic, for reasons outlined below. The key constraint on the synaptic plasticity rules within a connection is that the net connection weight (resulting from the combination of the average inhibitory and excitatory conductances within a connection) is adjusted as predicted by the rate-based BCM rule (Equation 23). In the delta rule network this means that when the post-synaptic rate is near *r*_θ_ (Figure 2B) and the net connection weight increases, the synaptic rules should produce a net decrease in this weight. This does not preclude the excitatory synapses from being potentiated, but it does mean that the inhibitory synapses should be potentiated *more*. In this sense we say that net plasticity will be anti-Hebbian. We restricted ourselves to only using only one type of plastic synapse to minimize the number of parameters. The evidence for anti-Hebbian rules is sparse for excitatory synapses (although see Sjöström and Häusser, 2006 and Letzkus et al., 2006), but is present for inhibitory synapses (Haas et al., 2006). Additionally, prior theoretical work concerning probabilistic inference also suggests the use of anti-Hebbian plasticity in excitatory and inhibitory synapses (Rezende et al., 2011). Unlike that work, however, our model predicts anti-Hebbian plasticity in both top-down and bottom-up connections.

Consistent with the ideas presented in this work, the kernels in the inhibitory synaptic plasticity rule found in the Entorhinal cortex of the rat by Haas et al. (2006) show a pronounced dip for near-coincident pre- and post-synaptic spikes. The STDPi rule proposed in this paper goes beyond the experimental data as it posits a BCM-like quadratic post-synaptic rate dependence (Figure 2B), something that was not explored in the experiments. The experimental plasticity rule also has time constants that are shorter than the well-performing STDPi kernels require, but this may be explained by the biological neurons in the experiments having cell dynamics that operate on a faster time scale than those of our model neurons. We predict that brain areas which implement the rate-based wake-sleep algorithm that we presented here will have adaptations (in the form of kernel shape or the timing of the weight changes) to reduce the bias introduced by spike correlations.

While our formalism is based on connections with net anti-Hebbian plasticity, our model does not require that all connections in the brain should be net anti-Hebbian. Non-hierarchical generative models such as those with lateral connections within a layer may require alternate plasticity rules to learn those connection weights (Dayan, 1999). Alternatively, not every part of the brain may require an explicit generative model, and thus be better described by other, non-Helmholtz Machine, frameworks (Brea et al., 2011; Nessler et al., 2013). Overall, our proposal is compatible with the abundance of known Hebbian plasticity rules in the cortex and Hippocampus.

The wake-sleep algorithm is implemented in our model by rewiring the network for each phase. Such rewiring, however, need not be implemented in the brain via explicit silencing or shunting of synapses. For example, the connection between pool *O* and the output pool *X*_1_ depicted on Figure 4 could be “turned off” by strongly inhibiting the cells in pool *O* without any reconfiguration of connectivity. This inhibition can be periodic, which is consistent with the abundance of rhythms in the cortex (Buzsáki and Draguhn, 2004), although such clock-like periodicity is not required for the wake-sleep algorithm to function.

The wake and sleep phases may correspond to the actual wakefulness and sleep of an animal. There is evidence of circadian fluctuation of modulators that affect learning (Steriade, 2004; Welberg, 2013) and corresponding changes in the observed firing patterns of neurons (Sherman, 2001) and overall functional connectivity (Massimini et al., 2005). Alternatively, rapid perceptual learning can happen without intervening sleep (Hawkey et al., 2004; Alain et al., 2007), which suggests that the wake and sleep phases may correspond to the state of the network when a relevant stimulus is present (and attended to), while the sleep phase represents the spontaneous state of the network (or a state of inattention). The required connectivity switches would then be caused by the different dynamics of the network in states with differing levels of attentiveness. Such attention dependent dynamics have been observed in a number of sensory cortices (Fontanini and Katz, 2006, 2008).

Our networks utilize sparse random connections between pools, but include no homeostatic and structural plasticity mechanisms to adjust the non-plastic connections to counteract unfavorable realizations of random connectivity. In the worst case scenarios, a neuron may be entirely disconnected from upstream neurons, or be tonically active. This contributes to the great variability in performance between different network realizations, and an overall suboptimal performance compared to the computational Hemlholtz machine. We believe the, in contrast, near-optimal animal behavior stems from the brain utilizing such mechanisms (Holtmaat and Svoboda, 2009; Vitureira et al., 2012), which, if added to our models, would likely serve to close the apparent gap in average performance of our networks and experiments.

Our model uses a very simplistic coding strategy to represent continuous variables, with the mean rate of a population of neurons exactly interpretable as the value of a variable. One consequence of this is that the variance of the encoded probability distribution of a variable depends inversely on the number of neurons used to code it. By using relatively small neuronal pools, we assure a high amount of variance. When this is detrimental to a task (e.g., Figure 13) we expect the brain to pool the activities of multiple unit networks in order to decrease the variance in the decision output.

One further issue that arises from our encoding strategy is its difficulty in representing negative weights, which caused the neuronal networks to have trouble modeling probability distributions with negative correlations. Rather than a one-to-one correspondence between firing rates and stimulus variables, the use of a more sophisticated coding strategy (e.g., using ideas from Eliasmith and Anderson, 2003) within the Helmholtz Machine framework is a natural extension of our work that would resolve such issues.

Throughout the paper we have represented samples from the probability distribution as being the mean rate of a pool of neurons over 500 ms. This is inconsistent with data that shows that correlations between the activity of different neurons decay over 20–40 ms (Berkes et al., 2011) and that perceptual decisions can be made on a similarly short timescale (Stanford et al., 2010). The duration of each sample necessary for successful learning in our implementation depends critically on the timescale that the synaptic plasticity rules use to estimate the relevant rates. In our preliminary modeling we have observed (data not shown) that the learning performance of the networks drops markedly when the samples are reduced in duration (the shortest sample duration we tested was 100 ms long). The reduction was more pronounced for the STDPi rule than the Spiking BCM rule, consistent with the overall reduced performance of the former shown in this paper. These issues, however, should only arise during training. Outside of training shorter samples can be used, thus allowing the framework to model fast inferences.

Overall, we think that the approach taken in this paper to implement the Helmholtz Machine in a neuronal network is promising and improvements to the model along the possible directions discussed above may provide a unified explanation for how probabilistic inference is performed in the brain.

## Author contributions

PS and PM designed the experiments and models. PS wrote the manuscript, performed the simulations, collected data and conducted the analyses.

### Conflict of interest statement

The authors declare that the research was conducted in the absence of any commercial or financial relationships that could be construed as a potential conflict of interest.

## References

[B1] Alain C. Snyder J. S. He Y. Reinke K. S. (2007). Changes in auditory cortex parallel rapid perceptual learning. Cereb. Cortex 17, 1074–1084. 10.1093/cercor/bhl018 16754653

[B2] Alais D. Burr D. (2004). The ventriloquist effect results from near-optimal bimodal integration. Curr. Biol. 14, 257–262. 10.1016/j.cub.2004.01.029 14761661

[B3] Alexandrescu A. (2010). The D Programming Language. Boston, MA: Addison-Wesley Professional.

[B4] Atkins J. E. Fiser J. Jacobs R. A. (2001). Experience-dependent visual cue integration based on consistencies between visual and haptic percepts. Vis. Res. 41, 449–461. 10.1016/S0042-6989(00)00254-6 11166048

[B5] Berkes P. Orbán G. Lengyel M. Fiser J. (2011). Spontaneous cortical activity reveals hallmarks of an optimal internal model of the environment. Science 331, 83–87. 10.1126/science.1195870 21212356 PMC3065813

[B6] Bienenstock E. L. Cooper L. N. Munro P. W. (1982). Theory for the development of neuron selectivity: orientation specificity and binocular interaction in visual cortex. J. Neurosci. 2, 32–48. 7054394 10.1523/JNEUROSCI.02-01-00032.1982 PMC6564292

[B7] Bishop C. M. (2006). Pattern Recognition and Machine Learning. New York, NY: Springer.

[B8] Blaisdell A. P. Sawa K. Leising K. J. Waldmann M. R. (2006). Causal reasoning in rats. Science 311, 1020–1022. 10.1126/science.1121872 16484500

[B9] Brea J. Senn W. Pfister J. (2011). Sequence learning with hidden units in spiking neural networks. Adv. Neural Inf. Process. Syst. 24, 1422–1430.

[B10] Buesing L. Bill J. Nessler B. Maass W. (2011). Neural dynamics as sampling: a model for stochastic computation in recurrent networks of spiking neurons. PLoS Comput. Biol. 7:e1002211. 10.1371/journal.pcbi.1002211 22096452 PMC3207943

[B11] Burge J. Girshick A. R. Banks M. S. (2010). Visual-haptic adaptation is determined by relative reliability. J. Neurosci. 30, 7714–7721. 10.1523/JNEUROSCI.6427-09.2010 20519546 PMC3056491

[B12] Buzsáki G. Draguhn A. (2004). Neuronal oscillations in cortical networks. Science 304, 1926–1929. 10.1126/science.1099745 15218136

[B13] Castillo J. D. Katz B. (1954). Quantal components of the end-plate potential. J. Physiol. 108, 783–794. 13175199 10.1113/jphysiol.1954.sp005129 PMC1366292

[B14] Chalk M. Seitz A. Seriès P. (2010). Rapidly learned stimulus expectations alter perception of motion. J. Vis. 10, 1–18. 10.1167/10.8.2.Introduction 20884577

[B15] Dayan P. Hinton G. E. Neal R. M. Zemel R. S. (1995). The Helmholtz machine. Neural Comput. 7, 889–904. 7584891 10.1162/neco.1995.7.5.889

[B16] Dayan P. (1999). Recurrent sampling models for the Helmholtz machine. Neural Comput. 11, 653–677. 10085425 10.1162/089976699300016610

[B17] Dayan P. (2000). Helmholtz machines and wake-sleep learning, in Handbook of Brain Theory and Neural Network, ed Arbib M. (Cambridge, MA: MIT Press), 44.

[B18] Deneve S. (2008a). Bayesian spiking neurons I: inference. Neural Comput. 20, 91–117. 10.1162/neco.2008.20.1.91 18045002

[B19] Deneve S. (2008b). Bayesian spiking neurons II: learning. Neural Comput. 20, 118–145. 10.1162/neco.2008.20.1.118 18045003

[B20] D'Souza P. Liu S.-C. Hahnloser R. H. R. (2010). Perceptron learning rule derived from spike-frequency adaptation and spike-time-dependent plasticity. Proc. Natl. Acad. Sci. U.S.A. 107, 4722–4727. 10.1073/pnas.0909394107 20167805 PMC2842046

[B21] Eliasmith C. Anderson C. H. (2003). Neural Engineering: Computation, Representation, and Dynamics in Neurobiological Systems, Vol. 15 of Computational Neuroscience. Cambridge, MA: MIT Press.

[B22] Ernst M. O. Banks M. S. (2002). Humans integrate visual and haptic information in a statistically optimal fashion. Nature 415, 429–433. 10.1038/415429a 11807554

[B23] Feng S. Holmes P. Rorie A. Newsome W. T. (2009). Can monkeys choose optimally when faced with noisy stimuli and unequal rewards? PLoS Comput. Biol. 5:e1000284. 10.1371/journal.pcbi.1000284 19214201 PMC2631644

[B24] Fiser J. Berkes P. Orbán G. Lengyel M. (2010). Statistically optimal perception and learning: from behavior to neural representations. Trends Cogn. Sci. 14, 119–130. 10.1016/j.tics.2010.01.003 20153683 PMC2939867

[B25] Fontanini A. Katz D. B. (2006). State-dependent modulation of time-varying gustatory responses. J. Neurophysiol. 96, 3183–3193. 10.1152/jn.00804.2006 16928791

[B26] Fontanini A. Katz D. B. (2008). Behavioral states, network states, and sensory response variability. J. Neurophysiol. 100, 1160–1168. 10.1152/jn.90592.2008 18614753 PMC2544460

[B27] Friston K. Kiebel S. (2009). Cortical circuits for perceptual inference. Neural Netw. 22, 1093–1104. 10.1016/j.neunet.2009.07.023 19635656 PMC2796185

[B28] Griffiths T. L. Tenenbaum J. B. (2006). Optimal predictions in everyday cognition. Psychol. Sci. 17, 767–773. 10.1111/j.1467-9280.2006.01780.x 16984293

[B29] Griffiths T. L. Chater N. Kemp C. Perfors A. Tenenbaum J. B. (2010). Probabilistic models of cognition: exploring representations and inductive biases. Trends Cogn. Sci. 14, 357–364. 10.1016/j.tics.2010.05.004 20576465

[B30] Haas J. S. Nowotny T. Abarbanel H. D. I. (2006). Spike-timing-dependent plasticity of inhibitory synapses in the entorhinal cortex. J. Neurophysiol. 96, 3305–3313. 10.1152/jn.00551.2006 16928795

[B31] Han F. Caporale N. Dan Y. (2008). Reverberation of recent visual experience in spontaneous cortical waves. Neuron 60, 321–327. 10.1016/j.neuron.2008.08.026 18957223 PMC3576032

[B32] Hancock P. J. B. Smith L. S. Phillips W. A. (1991). A biologically supported error-correcting learning rule. Neural Comput. 3, 201–212. 10.1162/neco.1991.3.2.201 31167306

[B33] Hawkey D. J. C. Amitay S. Moore D. R. (2004). Early and rapid perceptual learning. Nat. Neurosci. 7, 1055–1056. 10.1038/nn1315 15361880

[B34] Helmholtz H. (1925). Treatise on Physiological Optics, 3rd Edn Rochester, NY: The Optical Society of America.

[B35] Hinton G. E. Dayan P. (1996). Varieties of Helmholtz machine. Neural Netw. 9, 1385–1403. 12662541 10.1016/s0893-6080(96)00009-3

[B36] Hinton G. E. Dayan P. Frey B. J. Neal R. M. (1995). The “wake-sleep” algorithm for unsupervised neural networks. Science 268, 1158–1161. 7761831 10.1126/science.7761831

[B37] Holtmaat A. Svoboda K. (2009). Experience-dependent structural synaptic plasticity in the mammalian brain. Nat. Rev. Neurosci. 10, 647–658. 10.1038/nrn2699 19693029

[B38] Izhikevich E. M. (2003). Simple model of spiking neurons. IEEE Trans. Neural Netw. 14, 1569–1572. 10.1109/TNN.2003.820440 18244602

[B39] Jahr C. Stevens C. (1990). Voltage dependence of NMDA-activated macroscopic conductances predicted by single-channel kinetics. J. Neurosci. 10, 3178–3182. 1697902 10.1523/JNEUROSCI.10-09-03178.1990 PMC6570236

[B40] Jones E. Oliphant T. Peterson P. (2001). SciPy: Open Source Scientific Tools for Python. Available online at: http://www.scipy.org/scipylib/citing.html (Accessed October 23, 2013).

[B41] Körding K. P. Wolpert D. M. (2004). Bayesian integration in sensorimotor learning. Nature 427, 244–247. 10.1038/nature02169 14724638

[B42] Kullback S. Leibler R. A. (1951). On information and sufficiency. Ann. Math. Stat. 22, 79–86.

[B43] Lee T. S. (2002). Top-down influence in early visual processing: a Bayesian perspective. Physiol. Behav. 77, 645–650. 10.1016/S0031-9384(02)00903-4 12527013

[B44] Letzkus J. J. Kampa B. M. Stuart G. J. (2006). Learning rules for spike timing-dependent plasticity depend on dendritic synapse location. J. Neurosci. 26, 10420–10429. 10.1523/JNEUROSCI.2650-06.2006 17035526 PMC6674691

[B45] Lin J. (1991). Divergence measures based on the Shannon entropy. IEEE Trans. Inform. Theory 37, 145–151.

[B46] Lochmann T. Deneve S. (2011). Neural processing as causal inference. Curr. Opin. Neurobiol. 21, 774–781. 10.1016/j.conb.2011.05.018 21742484

[B47] Ma W. J. Beck J. M. Latham P. E. Pouget A. (2006). Bayesian inference with probabilistic population codes. Nat. Neurosci. 9, 1432–1438. 10.1038/nn1790 17057707

[B48] Ma W. J. Beck J. M. Pouget A. (2008). Spiking networks for Bayesian inference and choice. Curr. Opin. Neurobiol. 18, 217–222. 10.1016/j.conb.2008.07.004 18678253

[B49] Massimini M. Ferrarelli F. Huber R. Esser S. K. Singh H. Tononi G. (2005). Breakdown of cortical effective connectivity during sleep. Science 309, 2228–2232. 10.1126/science.1117256 16195466

[B50] Moran R. J. Campo P. Symmonds M. Stephan K. E. Dolan R. J. Friston K. J. (2013). Free energy, precision and learning: the role of cholinergic neuromodulation. J. Neurosci. 33, 8227–8236. 10.1523/JNEUROSCI.4255-12.2013 23658161 PMC4235126

[B51] Neal R. M. Dayan P. (1997). Factor analysis using delta-rule wake-sleep learning. Neural Comput. 9, 1781–1803. 9377277 10.1162/neco.1997.9.8.1781

[B52] Nessler B. Pfeiffer M. Buesing L. Maass W. (2013). Bayesian computation emerges in generic cortical microcircuits through spike-timing-dependent plasticity. PLoS Comput. Biol. 9:e1003037. 10.1371/journal.pcbi.1003037 23633941 PMC3636028

[B53] Okun M. Yger P. Marguet S. L. Gerard-Mercier F. Benucci A. Katzner S.. (2012). Population rate dynamics and multiNeuron firing patterns in sensory cortex. J. Neurosci. 32, 17108–17119. 10.1523/JNEUROSCI.1831-12 23197704 PMC3520056

[B54] Orbán G. Fiser J. Aslin R. N. R. Lengyel M. (2008). Bayesian learning of visual chunks by human observers. Proc. Natl. Acad. Sci. U.S.A. 105, 2745–2750. 10.1073/pnas.0708424105 18268353 PMC2268207

[B55] Pecevski D. Buesing L. Maass W. (2011). Probabilistic inference in general graphical models through sampling in stochastic networks of spiking Neurons. PLoS Comput. Biol. 7:e1002294 10.1371/journal.pcbi.1002294 22219717 PMC3240581

[B56] Pfister J.-P. Gerstner W. (2006). Triplets of spikes in a model of spike timing-dependent plasticity. J. Neurosci. 26, 9673–9682. 10.1523/JNEUROSCI.1425-06.2006 16988038 PMC6674434

[B57] Rao R. (2005). Hierarchical Bayesian Inference in Networks of Spiking Neurons, Vol. 17 Cambridge, MA: MIT Press.

[B58] Rezende D. Wierstra D. Gerstner W. (2011). Variational learning for recurrent spiking networks. Adv. Neural Inf. Process. Syst. 24, 136–144. 24772078

[B59] Sanborn A. N. Griffiths T. L. Navarro D. J. (2010). Rational approximations to rational models: alternative algorithms for category learning. Psychol. Rev. 117, 1144–1167. 10.1037/a0020511 21038975

[B60] Sherman S. M. (2001). Tonic and burst firing: dual modes of thalamocortical relay. Trends Neurosci. 24, 122–126. 10.1016/S0166-2236(00)01714-8 11164943

[B61] Shi L. Griffiths T. (2009). Neural implementation of hierarchical Bayesian inference by importance sampling. Adv. Neural Inf. Process. Syst. 22, 1669–1677.

[B62] Sjöström P. J. Häusser M. (2006). A cooperative switch determines the sign of synaptic plasticity in distal dendrites of neocortical pyramidal Neurons. Neuron 51, 227–238. 10.1016/j.neuron.2006 16846857 PMC7616902

[B63] Stanford T. R. Shankar S. Massoglia D. P. Costello M. G. Salinas E. (2010). Perceptual decision making in less than 30 milliseconds. Nat. Neurosci. 13, 379–385. 10.1038/nn.2485 20098418 PMC2834559

[B64] Steriade M. (2004). Acetylcholine systems and rhythmic activities during the waking–sleep cycle. Prog. Brain Res. 145, 179–196. 10.1016/S0079-6123(03)45013-9 14650916

[B65] Stone J. Gohara D. Shi G. (2010). OpenCL: a parallel programming standard for heterogeneous computing systems. Comput. Sci. Eng. 12, 66–72. 10.1109/MCSE.2010.69 21037981 PMC2964860

[B66] Sutherland G. R. McNaughton B. (2000). Memory trace reactivation in hippocampal and neocortical neuronal ensembles. Curr. Opin. Neurobiol. 10, 180–186. 10.1016/S0959-4388(00)00079-9 10753801

[B67] Tassinari H. Hudson T. E. Landy M. S. (2006). Combining priors and noisy visual cues in a rapid pointing task. J. Neurosci. 26, 10154–10163. 10.1523/JNEUROSCI.2779-06 17021171 PMC6674625

[B68] Treves A. Panzeri S. (1995). The upward bias in measures of information derived from limited data samples. Neural Comput. 7, 399–407 10.1162/neco.1995.7.2.399

[B69] Turrigiano G. G. Nelson S. B. (2004). Homeostatic plasticity in the developing nervous system. Nat. Rev. Neurosci. 5, 97–107. 10.1038/nrn1327 14735113

[B70] van Beers R. J. Sittig A. C. Gon J. J. (1999). Integration of proprioceptive and visual position-information: an experimentally supported model. J. Neurophysiol. 81, 1355–1364. 10085361 10.1152/jn.1999.81.3.1355

[B71] van Rossum G. Drake F. L. (2001). Python Reference Manual. Pythonlabs. Available online at: http://www.python.org

[B72] Vitureira N. Letellier M. Goda Y. (2012). Homeostatic synaptic plasticity: from single synapses to neural circuits. Curr. Opin. Neurobiol. 22, 516–521. 10.1016/j.conb.2011.09.006 21983330 PMC3378479

[B73] Weiss Y. Simoncelli E. P. Adelson E. H. (2002). Motion illusions as optimal percepts. Nat. Neurosci. 5, 598–604. 10.1038/nn0602-858 12021763

[B74] Welberg L. (2013). Learning and memory: learning with peaks and troughs. Nat. Rev. Neurosci. 14, 380–381. 10.1038/nrn3515 23657555

