# Supplementary material for: Spiking neuron network Helmholtz machine
Source: Front Comput Neurosci. 2015 Apr 21;9:46. doi: 10.3389/fncom.2015.00046 (PMC4405618; doi:10.3389/fncom.2015.00046)
Supplement: Supplementary file 1 [file Presentation1.PDF]

Frontiers Computational Neuroscience

*Research Article*
*10 April 2015*

# *Supplementary Material*: Spiking Neuron Network Helmholtz Machine

**Pavel Sountsov [1,2,*] and Paul Miller [2,3]**

[1] *Neuroscience Graduate Program, Brandeis University, Waltham, MA, USA*
[2] *Volen National Center for Complex Systems, Brandeis University, Waltham, MA, USA*
[3] *Department of Biology, Brandeis University, Waltham, MA, USA*

Correspondence*:
Pavel Sountsov
MS 008 415 South St Brandeis University P.O. Box 549110 Waltham, MA 02454-9110, sl157@brandeis.edu

## STOCHASTIC SAMPLING OUTPUT OF THE DELTA RULE NETWORK

To examine whether the delta rule network stochastically samples from some conditional probability distribution, we simulated it with plasticity disabled for 1000 simulated seconds with a fixed input rate ($r_E$) of 30 Hz and examined the activity of the output pool O (see Figure 2). Two seconds of the simulated spiking activity of the two pools are shown in Figure S1A and B. The spikes of the neurons in those pools were binned in 500 ms bins, and averaged across each pool to produce putative samples from a probability distribution. The joint probability distribution of the mean rates of the two pools is shown in Figure S1C. By taking a slice of that joint probability (for $r_E$ = 30 Hz), we produce the conditional probability distribution of the delta rule network (at that input rate), as shown in Figure S1D.

## THE LEARNING RULE FOR LINEAR TRUNCATED GAUSSIAN UNITS

To derive the weight adaptation rule for the $i$'th component of the weight vector $\mathbf{w}$ for a linear truncated gaussian unit, we need to compute

$$\frac{\partial}{\partial w} \log \mathcal{N}(z; \mathbf{w}^T\mathbf{x} + b, \sigma) = \frac{\partial}{\partial w} \log \mathcal{N}(z),$$

where $b$ is a bias value (**Hinton and Dayan**, 1996). Plugging in the definition for $\mathcal{N}(\cdot)$ (Equation 2), and taking advantage of the fact that $z \geq 0$ we see that this reduces to:

$$\begin{aligned}
\frac{\partial}{\partial w} \log \mathcal{N}(z) &= \frac{\partial}{\partial w} (-\log Z + \log H(z) + \log N(z)) \\
&= 0 + \frac{\partial}{\partial w} (0 + \log N(z)) \\
&= \frac{\partial}{\partial w} \log N(z) \\
&\propto x_i(z - (\mathbf{w}^T\mathbf{x} + b)).
\end{aligned}$$

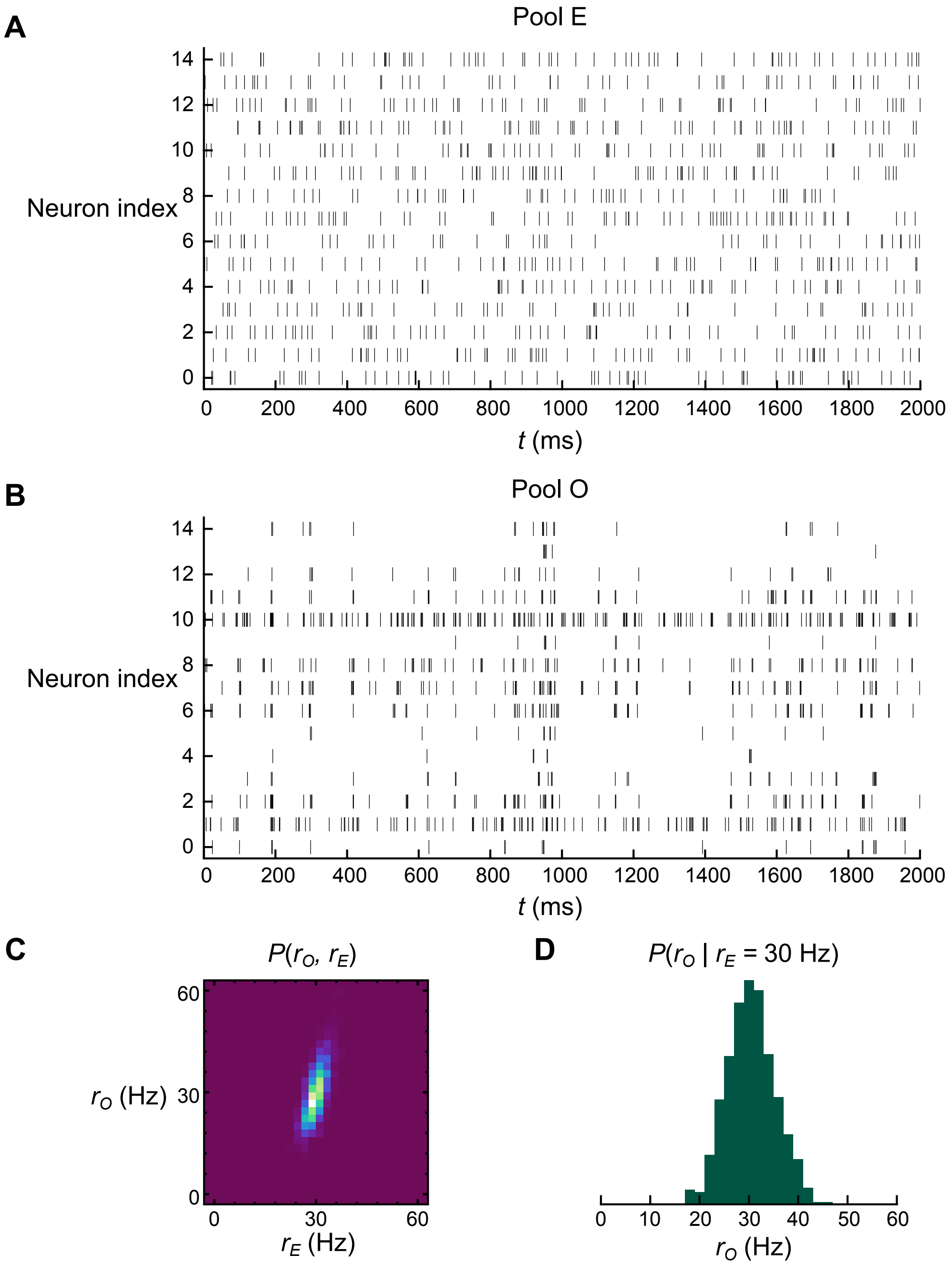


**Figure S1.** Stochastic activity of a delta rule network (see Figure 2). A) The spiking activity of the neurons inside input pool E, given a set rate of 30 Hz. B) The spiking activity of the neurons inside output pool O, corresponding to the activity in panel A. The initial total weight of the plastic connections was set to 1.24 nS. C) The joint probability distribution of the mean rates (across the neurons inside a pool) of the the input and output pools. The rates were computed in 500 ms bins, for a total of 2000 bins. The variability in the input pool's mean rate is entirely due to the Poisson spiking of the individual neurons in that pool. D) Conditional probability distribution of the mean rate of the output pool given that the mean rate of the input is 30 Hz.

The derivation of the bias adaptation rule is analogous.

## SPIKING PLASTICITY RULES AND THE BCM RULE

Here we show how the proposed synaptic plasticity rules implement the fixed threshold, rate-based BCM rule for uncorrelated Poisson pre- and post-synaptic spike trains using standard methods (**Dayan and Abbott**, 2001).

First, let us restate the definitions of the two spiking rules we consider (also see Methods), as well as the rate-based BCM rule. For the BCM rule, the weight is updated during each pre-synaptic spike,

$$w \mapsto w + A\hat{r}_{post}(t_i)(\hat{r}_{post}(t_i) - r_\theta), \tag{S1}$$

where

$$\hat{r}_{post}(t) = \sum_j \kappa(t - t_j)$$
$$\kappa(t) = \begin{cases} 0 & \text{if } t < 0 \\ \frac{1}{\tau}\exp\left(-\frac{t}{\tau}\right) & \text{if } t \geq 0 \end{cases}. \tag{S2}$$

For the STDPi rule, the weight is updated both during the pre-synaptic spike,

$$w \mapsto w - A_m\hat{r}_{post}(t_i), \tag{S3}$$

and post-synaptic spike,

$$w \mapsto w + A_p\hat{r}_{pre}(t_j)\hat{r}_{post}(t_j), \tag{S4}$$

where

$$\hat{r}_{pre}(t) = \sum_i \kappa(t - t_i)$$
$$\hat{r}_{post}(t) = \sum_j \kappa(t - t_j)$$
$$\kappa(t) = \begin{cases} 0 & \text{if } t < 0 \\ \frac{1}{\tau_1 - \tau_2}\left(\exp\left(-\frac{t}{\tau_1}\right) - \exp\left(-\frac{t}{\tau_2}\right)\right) & \text{if } t \geq 0 \end{cases}.$$

The rate-based BCM rule we are trying to match with these spike based plasticity rules is:

$$\frac{dw}{dt} = \eta r_{pre} r_{post}(r_{post} - r_\theta). \tag{S5}$$

Starting with the Spiking BCM rule, we first compute the expected value (across all post-synaptic spike trains) of the weight change detailed in Equation S1. Expanding the product and taking the expectation we obtain the following expression:

$$\langle \Delta w \rangle = A \left\langle \hat{r}_{post}^2 \right\rangle - A \left\langle \hat{r}_{post} \right\rangle r_\theta. \tag{S6}$$

Starting with the second expectation, we first use the kernel definition (Equation S2) to express how the value of $\hat{r}_{post}$ changes in response to a new post-synaptic spike given the time that passed since the last spike $\Delta t_j = t_j - t_{j-1}$:

$$\hat{r}_{post}\left(t_j^-\right) = \hat{r}_{post}\left(t_{j-1}^+\right) \exp\left(\frac{-\Delta t_j}{\tau}\right)$$
$$\hat{r}_{post}\left(t_j^+\right) = \hat{r}_{post}\left(t_j^-\right) + \frac{1}{\tau},$$

where $t_j^-$ and $t_j^+$ are the times just before and just after $j$ 'th post-synaptic spike while $t_{j-1}^+$ is the time just after $j-1$ 'th post-synaptic spike. Next we compute the expectation over the above expressions. In doing so, we take advantage of the fact that the Poisson process that generates the spikes is memoryless, which means that each pair of successive spikes can be examined independently. Additionally, the interval between the spikes does not depend on the absolute time of the two spikes. We thus write

$$\left\langle \hat{r}_{post}\left(t^-\right) \right\rangle = \left\langle \hat{r}_{post}\left(t^+\right) \right\rangle \left\langle \exp\left(\frac{-\Delta t}{\tau}\right) \right\rangle \tag{S7}$$

$$\left\langle \hat{r}_{post}\left(t^+\right) \right\rangle = \left\langle \hat{r}_{post}\left(t^-\right) \right\rangle + \frac{1}{\tau} \tag{S8}$$

The expectation of the kernel trace can be computed directly using the definition of $P(\Delta t)$ of a Poisson process with rate $r_{post}$:

$$\begin{aligned}\left\langle \exp\left(\frac{-\Delta t}{\tau}\right) \right\rangle &= \int_0^\infty P(\Delta t) \exp\left(\frac{-\Delta t}{\tau}\right) d\Delta t \\ &= \int_0^\infty r_{post} \exp\left(-r_{post}\Delta t\right) \exp\left(\frac{-\Delta t}{\tau}\right) d\Delta t \\ &= \frac{r_{post}\tau}{r_{post}\tau + 1}.\end{aligned} \tag{S9}$$

We now substitute Equation S9 into Equation S7 and then use Equation S8 to find that:

$$\left\langle \hat{r}_{post}\left(t^-\right) \right\rangle = r_{post}.$$

Now we also compute $\langle \hat{r}_{post} \rangle$ by computing the expected average kernel trace between two post-synaptic spikes:

$$\begin{aligned}
\langle \hat{r}_{post} \rangle &= \frac{1}{\langle \Delta t \rangle} \left\langle \int_0^{\Delta t} \hat{r}_{post}\left(t^+\right) \exp\left(\frac{-t}{\tau}\right) \, dt \right\rangle \\
&= r_{post} \left\langle \tau \left( \hat{r}_{post}\left(t^+\right) - \hat{r}_{post}\left(t^+\right) \exp\left(\frac{-t}{\tau}\right)\right)\right\rangle \\
&= r_{post}\tau \left( \left\langle \hat{r}_{post}\left(t^+\right)\right\rangle - \left\langle \hat{r}_{post}\left(t^-\right)\right\rangle \right) \\
&= r_{post}\tau \left(\frac{1}{\tau}\right) \\
&= r_{post}.
\end{aligned} \tag{S10}$$

Computing the first expectation of Equation S6 using the same technique we find that

$$\begin{aligned}
\left\langle \hat{r}^2_{post} \right\rangle &= \frac{1}{\langle \Delta t \rangle} \left\langle \int_0^{\Delta t} \hat{r}^2_{post}\left(t^+\right) \exp\left(\frac{-2t}{\tau}\right) \, dt \right\rangle \\
&= r_{post} \left\langle \frac{\tau}{2} \left( \hat{r}^2_{post}\left(t^+\right) - \hat{r}^2_{post}\left(t^+\right) \exp\left(\frac{-2\Delta t}{\tau}\right)\right)\right\rangle \\
&= r_{post}\frac{\tau}{2} \left( \left\langle \hat{r}^2_{post}\left(t^+\right)\right\rangle - \left\langle \hat{r}^2_{post}\left(t^-\right)\right\rangle \right) \\
&= r_{post}\frac{\tau}{2} \left(\frac{2}{\tau}\left\langle \hat{r}_{post}\left(t^-\right)\right\rangle + \frac{1}{\tau^2}\right) \\
&= r^2_{post} + \frac{1}{2\tau} r_{post}.
\end{aligned} \tag{S11}$$

We now can compute $\left\langle \frac{dw}{dt} \right\rangle$. As the weight changes happen during every pre-synaptic spike and the pre-synaptic spikes happen at a rate $r_{pre}$, we see that

$$\begin{aligned}
\left\langle \frac{dw}{dt} \right\rangle &= r_{pre} \langle \Delta w \rangle \\
&= A r_{pre} \left( r^2_{post} + \frac{1}{2\tau} r_{post} - r_{post} r_\theta \right) \\
&= A r_{pre} r_{post} \left( r_{post} - \frac{2\tau r_\theta - 1}{2\tau r_\theta} \right),
\end{aligned}$$

which is the same functional form as Equation S5. Additionally, the theoretical location of the sign change is consistent with the computational studies (Figure 3D, left).

To prove that STDPi implements the BCM rule, it is convenient to split its kernel into two exponential kernels that each provides an estimate of the rate,

$$\kappa_1(t) = \begin{cases} 0 & \text{if } t < 0 \\ \frac{1}{\tau_1} \exp\left(-\frac{t}{\tau_1}\right) & \text{if } t \geq 0 \end{cases}$$

$$\kappa_2(t) = \begin{cases} 0 & \text{if } t < 0 \\ \frac{1}{\tau_2} \exp\left(-\frac{t}{\tau_2}\right) & \text{if } t \geq 0 \end{cases}$$

$$\hat{r}_1(t) = \sum_i \kappa_1(t - t_i) \tag{S12}$$

$$\hat{r}_2(t) = \sum_i \kappa_2(t - t_i). \tag{S13}$$

The two rate estimates are then averaged to produce the rate estimate of the combined kernel:

$$\hat{r}(t) = \frac{\tau_1 \hat{r}_1(t) - \tau_2 \hat{r}_2(t)}{\tau_1 - \tau_2}.$$

We can then apply the same analysis as with the Spiking BCM rule on each of the two rate estimates to determine that $\langle \hat{r} \rangle = r$ in all locations where the estimate is needed in Equations 13 and 14. The resultant expression for the expected weight changes occurring during pre- and post-synaptic spikes is then:

$$\langle \Delta w_{pre} \rangle = -A_m r_{post}$$
$$\langle \Delta w_{post} \rangle = A_p r_{pre} r_{post}.$$

The rate at which those changes happen equals exactly $r_{pre}$ and $r_{post}$ respectively, so we can write the total rate of weight change as:

$$\begin{aligned} \left\langle \frac{dw}{dt} \right\rangle &= r_{pre} \left\langle \Delta w_{pre} \right\rangle + r_{post} \left\langle \Delta w_{post} \right\rangle \\ &= -A_m r_{pre} r_{post} + A_p r_{pre} r_{post}^2 \\ &= A_p r_{pre} r_{post} (r_{post} - \frac{A_m}{A_p}). \end{aligned}$$

This is the same functional form as Equation S5 and matches the computational studies (Figure 3D, right). Notably, unlike the Spiking BCM rule this equivalency does not depend on kernel shape. This occurs because unlike in the Spiking BCM rule, STDPi does not have any $\left\langle \hat{r}^2 \right\rangle$ terms which introduce the kernel shape dependency.

The short-term correlations between the pre- and post-synaptic spike trains introduced by post-synaptic potentials violate many of the assumptions made by the proofs above, and result in the synaptic plasticity rules no longer implementing the rate-based BCM rule. The first notable effect is that synaptic plasticity rules over-estimate $r_{post}$ in a weight dependent manner. To a first order approximation, this occurs due to the post-synaptic spike train becoming less Poisson-like (its CV grows to be greater than 1), which increases the value of $\left\langle \exp\left(\frac{-\Delta t}{\tau}\right) \right\rangle$ from Equation S7. In actuality, because the post-synaptic spike train

no longer has Poisson statistics (and is thus no longer memoryless) we cannot use Equations S7 and S8 to derive $\langle r_{post} \rangle$. The overestimation of $r_{post}$ is relatively small, and thus does appear to affect the functioning of our models.

The second notable effect, explored computationally in Figures 11 and 12, is that the STDPi rule underestimates the pre-synaptic rate. This manifests itself when Equation S10 is corrected to take into account the correlations (here we compute the expectation of the first pre-synaptic kernel trace of the STDPi rule),

$$\langle \hat{r}_1 \rangle = \frac{1}{Z} \left\langle \int_0^{\Delta t} \rho(t) r_1(t^+) \exp\left(-\frac{t}{\tau_1}\right) dt \right\rangle$$

$$Z = \left\langle \int_0^{\Delta t} \rho(t) \, dt \right\rangle ,$$

where $\frac{1}{Z}\rho(t)$ describes the probability of the post-synaptic spike occuring after a pre-synaptic spike (shifted to occur at $t = 0$).

## REFERENCES

Dayan, P. and Abbott, L. (2001), Theoretical Neuroscience (MIT Press)

Hinton, G. E. and Dayan, P. (1996), Varieties of Helmholtz Machine., *Neural networks : the official journal of the International Neural Network Society*, 9, 8, 1385–1403

## TABLES

**Table S1.** Parameters for the computational Helmholtz Machine model and synaptic plasticity rule exploration. $\mathbf{I}$ is a $2 \times 2$ identity matrix. $\mathbf{U}$ is a $2 \times 2$ matrix whose elements are sampled uniformly from $[0, 1]$.

| Parameter | Description | Value | Figures |
|---|---|---|---|
| $\beta$ | Bias rate | 100 Hz | 7, 9, 13, 14 |
| $\mathbf{\Sigma}$ | Variance of each unit | $4^2\mathbf{I}$ Hz | 7, 9, 13, 14 |
| $\mathbf{W_G}^{(0)}$ | Initial generative weights | $0.1\mathbf{U}$ | 7, 9, 13, 14 |
| $\mathbf{B_G}^{(0)}$ | Initial generative biases | $(0, 0)$ | 7, 9, 13, 14 |
| $\mathbf{W_R}^{(0)}$ | Initial recognition weights | $0.1\mathbf{U}$ | 7, 9, 13, 14 |
| $\mathbf{B_R}^{(0)}$ | Initial recognition biases | $(0, 0)$ | 7, 9, 13, 14 |
| $\eta$ | Learning rate | $2 \times 10^{-5}$ | 7, 9, 14 |
| | | $1 \times 10^{-7}$ | 13 |
| $N_{train}$ | Training duration | 500000 samples | 7, 9, 14 |
| | | 1000 – 50000 samples | 13 |
| $N_{sleep}$ | Extra sleep duration | 50000 samples | 13 |
| $N_{test}$ | Testing duration | 2000 samples | 7, 9, 13, 14 |
| 3 | | | |
| $N_{batch}$ | Number of consecutive samples in a single wake or sleep phase | 1 | 7, 9, 13, 14 |
| $\Delta r$ | Effect on $r_{post}(t)$ by a pre-synaptic spike | 0 Hz | 3 |
| | | $-40$ Hz | 11 |
| $\tau_r$ | Time constant of the effect on $r_{post}(t)$ by a pre-synaptic spike | 12 ms | 3, 11 |

**Table S2.** Parameters for neuron types. Neuron type abbreviations are: RS - Regular spiking (excitatory), FS - Fast spiking (inhibitory).

| Neuron Type | $a$ | $b$ | $c$ | $d$ |
|---|---|---|---|---|
| RS | 0.02 | 0.2 | -65 | 8 |
| FS | 0.1 | 0.2 | -65 | 2 |

**Table S3.** Parameters for neuronal pools. Neuron type abbreviations are: RS - Regular spiking (excitatory), FS - Fast spiking (inhibitory). Model abbreviations are: D - Delta rule network, HM - Hemlholtz Machine network.

| Pool | Neuron Type | Neuron Count | $r_r$ | $w_r$ | Model |
|---|---|---|---|---|---|
| $I$ | FS | 20 | 0 | 0 | D, HM |
| $T$ | RS | 15 | $r_T$ Hz | 7 nS | D, HM |
| $M$ | RS | 10 | 100 Hz | 0.42 nS | D, HM |
| $E$ | RS | 15 | $r_E$ Hz | 7 nS | D |
| $X_1, X_2, Y_1, Y_2$ | RS | 15 | 0 | 0 | HM |
| $D$ | FS | 15 | 0 | 0 | D, HM |
| $O$ | RS | 15 | 100 Hz | 0.6 nS | D, HM |
| $R$ | FS | 20 | 25 Hz | 5 nS | HM |

**Table S4.** Parameters for neuronal pools. Model abbreviations are: D - Delta rule network, HMW - Hemlholtz Machine network during wake phase, HMS - Hemlholtz Machine network during sleep phase. The total conductance refers to the sum of the conductances made by a single neuron in the source pool. Connections made from pools $X_1$, $X_2$, $Y_1$ and $Y_2$ cross units (e.g. the neurons from $X_1$ synapse onto neurons of pools $M$ associated with the two observed units $y_1$ and $y_2$).

| Source Pool | Destination Pool | Connection Type | Total Conductance | Model |
|---|---|---|---|---|
| $E$ | $I$ | Excitatory | 0.25 nS | D |
| $X_1, X_2, Y_1, Y_2$ | $I$ | Excitatory | 0.25 nS | HMW, HMS |
| $I$ | $M$ | Plastic Inhibitory | (varies) | D, HMW, HMS |
| $E$ | $M$ | Excitatory | 0.075 nS | D |
| $X_1, X_2, Y_1, Y_2$ | $M$ | Excitatory | 0.075 nS | HMW, HMS |
| $T$ | $M$ | Excitatory | 0.175 nS | D, HMW, HMS |
| $R$ | $M$ | Plastic Inhibitory | (varies) | HMW, HMS |
| $M$ | $D$ | Excitatory | 0.2 nS | D, HMW, HMS |
| $D$ | $O$ | Inhibitory | 2.2 nS | D, HMW, HMS |
| $T$ | $O$ | Excitatory | 0.6 nS | D, HMW, HMS |
| $O$ | $X_1, X_2$ | Excitatory | 0.375 nS | HMW |
| $O$ | $Y_1, Y_2$ | Excitatory | 0.375 nS | HMS |
| $T$ | $X_1, X_2$ | Excitatory | 0.375 nS | HMS |
| $T$ | $Y_1, Y_2$ | Excitatory | 0.375 nS | HMW |

**Table S5.** Parameters for the prior distributions. **I** is a $2 \times 2$ identity matrix. Model abbreviations are: C - Computational Model, B - Neuronal network with the Spiking BCM rule, S - Neuronal network with the STDPi rule.

| Parameter | Description | Value | Figures | Models |
|---|---|---|---|---|
| $\mu_{\mathbf{u}}$ | Location of the unimodal prior | $(15, 15)$ Hz | 7, 12 | C, B |
| | | $(45, 45)$ Hz | 8, 12 | S |
| $\mathbf{\Sigma_u}$ | Variance of the unimodal prior | $15^2\mathbf{I}$ Hz | 7, 12 | C, B |
| | | $45^2\mathbf{I}$ Hz | 8, 12 | S |
| $r_1$ | Mixing weight of the first blob of the bimodal prior | 0.5 | 9, 10, 12 | C, B, S |
| | | 0.5, 0.6, 0.7, 0.8 | 14 | C, B, S |
| $r_2$ | Mixing weight of the second blob of the bimodal prior | $1 - r_1$ | 9, 10, 12, 14 | C, B, S |
| $\mu_{\mathbf{b1}}$ | Location of the first blob of the bimodal prior | $(15, 0)$ Hz | 9, 12, 14 | C, B |
| | | $(45, 0)$ Hz | 10, 12, 14 | S |
| $\mu_{\mathbf{b2}}$ | Location of the second blob of the bimodal prior | $(0, 15)$ Hz | 9, 12, 14 | C, B |
| | | $(0, 45)$ Hz | 10, 12, 14 | S |
| $\mathbf{\Sigma_u}$ | Variance of each blob of the bimodal prior | $1\mathbf{I}$ Hz | 9, 12, 14 | C, B |
| | | $3^2\mathbf{I}$ Hz | 10, 12, 14 | S |

**Table S6.** Parameters for generative test training data sets.

| Data Set | $\theta$ | $\rho_u$ |
|---|---|---|
| $a$ | 0 | 5 Hz |
| $b$ | 0 | 15 Hz |
| $c$ | $\frac{\pi}{4}$ | 15 Hz |
| $d$ | $\frac{\pi}{2}$ | 15 Hz |
| $e$ | $\frac{3\pi}{4}$ | 15 Hz |
| $f$ | 0 | – |
| $g$ | $\frac{\pi}{4}$ | – |
| $h$ | $\frac{\pi}{2}$ | – |
| $i$ | $\frac{3\pi}{4}$ | – |

**Table S7.** Parameters for the neuronal networks. For parameters that vary between neuronal pools, see Table S3.

| Parameter | Description | Value | Figures |
|---|---|---|---|
| $r_{ns}$ | Non-specific input rate | 40 Hz | 7–10, 12, 13, 14 |
| $\Delta$ | Axonal delay | 1 ms | 6–10, 12, 13, 14 |
| $s$ | Connection sparsity | 0.3 | 6–10, 12, 13, 14 |
| $E_e$ | Reversal potential for excitatory synapses | 0 mV | 6–10, 12, 13, 14 |
| $E_i$ | Reversal potential for inhibitory synapses | $-80$ mV | 6–10, 12, 13, 14 |
| $[Mg^{++}]_{ext}$ | External concentration of $Mg^{++}$ | 1 mM | 6–10, 12, 13, 14 |
| $N_v$ | Number of vesicles per synapse | 10 | 6–10, 12, 13, 14 |
| $P_v$ | Probability of individual vesicle release | 0.25 | 6–10, 12, 13, 14 |
| $\tau_{e1}$ | Time constant of the AMPA conductance | 2 ms | 6–10, 12, 13, 14 |
| $\tau_{e2}$ | Time constant of the NMDA conductance | 50 ms | 6–10, 12, 13, 14 |
| $\tau_i$ | Time constant of the $GABA_A$ conductance | 10 ms | 6–10, 12, 13, 14 |

**Table S8.** Parameters for the synaptic plasticity rules.

| Parameter | Description | Value | Figures |
|---|---|---|---|
| $\tau$ | Time constant for the Spiking BCM kernel | $100\,\text{ms}$ | 3, 6, 7, 9, 13, 14 |
| $\tau_1$ | Long time constant for the STDPi kernel | $50\,\text{ms}$ | 3, 6, 8, 10, 13, 14 |
| $\tau_2$ | Short time constant for the STDPi kernel | $20\,\text{ms}$ | 3, 6, 8, 10, 13, 14 |
| | | $1, 30\,\text{ms}$ | 11 |
| | | $1, 5, 10, 15, 20, 25, 30\,\text{ms}$ | 12 |
| $A$ | Amplitude of weight change for the Spiking BCM rule | $4 \times 10^{-7}\,\text{Hz}^{-2}$ | 3 |
| | | $2 \times 10^{-6}\,\text{nS} \cdot \text{Hz}^{-2}$ | 6 |
| | | $1.922 \times 10^{-7}\,\text{nS} \cdot \text{Hz}^{-2}$ | 7, 9, 14 |
| | | $1.922 \times 10^{-9}\,\text{nS} \cdot \text{Hz}^{-2}$ | 13 |
| $A_m$ | Amplitude of weight depression for the STDPi rule | $1.125 \times 10^{-5}\,\text{Hz}^{-1}$ | 3 |
| | | $5.62 \times 10^{-5}\,\text{nS} \cdot \text{Hz}^{-1}$ | 6 |
| | | $5.4 \times 10^{-6}\,\text{nS} \cdot \text{Hz}^{-1}$ | 7, 9, 14 |
| | | $5.4 \times 10^{-8}\,\text{nS} \cdot \text{Hz}^{-1}$ | 13 |
| $A_p$ | Amplitude of weight potentiation for the STDPi rule | $4.17 \times 10^{-7}\,\text{Hz}^{-2}$ | 3 |
| | | $2.082 \times 10^{-6}\,\text{nS} \cdot \text{Hz}^{-2}$ | 6 |
| | | $2 \times 10^{-7}\,\text{nS} \cdot \text{Hz}^{-2}$ | 7, 9, 14 |
| | | $2 \times 10^{-9}\,\text{nS} \cdot \text{Hz}^{-2}$ | 13 |
| $r_\theta$ | Threshold rate for the Spiking BCM rule | $32\,\text{Hz}$ | 3, 6, 7, 9, 13, 14 |

**Table S9.** Training and testing parameters for the neuronal networks.

| Parameter | Description | Value | Figures |
|---|---|---|---|
| $T_{train}$ | Training duration for the delta rule network | 50 s | 6, 12 |
| $N_{train}$ | Training duration for the Helmholtz Machine network | 10000 samples | 7–10, 12 |
| | | 2000 – 10000 samples | 13 |
| | | 15000 samples | 14 |
| $N_{sleep}$ | Extra sleep duration | 1000 samples | 13 |
| $T_{test}$ | Testing duration for the delta rule network | 20 s | 6, 12A |
| $N_{test}$ | Testing duration for the Helmholtz Machine network | 2000 samples | 7, 8A,C,D, 9, 10, 12B, 13, 14 |
| | | 10000 samples | 8B |
| $T_{sample}$ | Sample duration | 500 ms | 7–10, 12–14 |
| $N_{batch}$ | Number of consecutive samples in a wake or sleep phase | 10 | 7–10, 12, 13 |
| | | 50 | 14 |
